# Interannual monsoon wind variability as a key driver of East African small pelagic fisheries

**DOI:** 10.1038/s41598-020-70275-9

**Published:** 2020-08-06

**Authors:** Fatma Jebri, Zoe L. Jacobs, Dionysios E. Raitsos, Meric Srokosz, Stuart C. Painter, Stephen Kelly, Michael J. Roberts, Lucy Scott, Sarah F. W. Taylor, Matthew Palmer, Hellen Kizenga, Yohana Shaghude, Juliane Wihsgott, Ekaterina Popova

**Affiliations:** 1 National Oceanography Centre, Southampton, SO14 3ZH UK; 2 Department of Biology, National and Kapodistrian University of Athens, Athens, Greece; 3 Plymouth Marine Laboratory, Plymouth, PL1 3DH UK; 4 Ocean Science and Marine Food Security, Nelson Mandela University, Port Elizabeth, 6001 South Africa; 5 South African Environmental Observation Network, Egagasini Node, Cape Town, South Africa; 6 Institute of Marine Sciences, Zanzibar, Tanzania

**Keywords:** Marine biology, Physical oceanography

## Abstract

Small pelagic fisheries provide food security, livelihood support and economic stability for East African coastal communities—a region of least developed countries. Using remotely- sensed and field observations together with modelling, we address the biophysical drivers of this important resource. We show that annual variations of fisheries yield parallel those of chlorophyll-a (an index of phytoplankton biomass). While enhanced phytoplankton biomass during the Northeast monsoon is triggered by wind-driven upwelling, during the Southeast monsoon, it is driven by two current induced mechanisms: coastal “dynamic uplift” upwelling; and westward advection of nutrients. This biological response to the Southeast monsoon is greater than that to the Northeast monsoon. For years unaffected by strong *El-Niño*/*La-Niña* events, the Southeast monsoon wind strength over the south tropical Indian Ocean is the main driver of year-to-year variability. This has important implications for the predictability of fisheries yield, its response to climate change, policy and resource management.

## Introduction

Several nations in the Western Indian Ocean (WIO) region face major socio-economic growth challenges such as the increasing need for livelihoods support and food security^1^ and high vulnerability to climate phenomena such as monsoons^2,3^. These countries are not only among the lowest ranked of the United Nations Human Development Index^4^, but are also highly dependent on fishing for economic stability and food security^5^. The *prevalence of severe food insecurity* indicator estimates 32.4% of the Eastern African population (~ 136.8 million people) were affected by undernourishment in 2017^6^. A case in point is Tanzania, where over two million people work in the fishing sector^7^ and fish supplies up to 70% of annual protein intake^8^. Small pelagic fish are particularly important for direct human consumption^9^, representing ~ 1/3 of the total marine catch with a diverse range of species including anchovies, shads, herrings, mackerel and sardines^10,11^. Although the fishing sector, dominated by artisanal and subsistence fisheries, has been under severe pressure due to increased exploitation^1^ and unsustainable practices^12^, environmental impacts are notable^13^, becoming the center of attention for government bodies in addressing the country’s challenges^14^. It is therefore of critical importance to develop a comprehensive understanding of physical and biogeochemical drivers that affect the long-term abundance of these small pelagic fish, particularly under the accelerating impact of climate change ^15^.

In contrast to many other parts of the global ocean, the Tanzanian seas are poorly studied. Nearly all existing studies have been *in-situ* based, reporting a surface circulation pattern (Supplementary Fig. S1) dominated by the East African Coastal Current (EACC), which is controlled by a monsoonal wind regime^16^. The EACC flows northward year-round^17^ with a change in intensity between seasons, being weakest in the Northeast monsoon (December to March) and strongest in the Southeast monsoon (May to October)^18,19^. The EACC originates as a branch of the South Equatorial Current (SEC), flowing from the central equatorial Indian Ocean towards Africa, where it diverges along the coast of Madagascar with a northern branch, the Northeast Madagascar Current (NEMC) and a southern one, the Southeast Madagascar Current (SEMC)^20^. The NEMC feeds the EACC.

Being of subtropical origins, Tanzanian waters are deemed to be relatively oligotrophic^18,21^ with low productivity^22^. Interestingly, the small pelagic fish potential yield within the Tanzanian Exclusive Economic Zone (EEZ) is estimated at 20,000 metric tons, which is ~ 1/3 that of Somalia, a highly productive upwelling area^23^. Moreover, the total marine landings grew by 50% between 1982 and 1993, partly through increased catches of small pelagics^24^. The unusually large small pelagic biomass found in this supposedly unproductive region raises several questions—one of which concerns the identification of key environmental factors that influence abundance.

Here, we investigate large-scale biophysical processes potentially affecting the herring, shad and anchovy populations (*Clupeidae* and *Engraulidae*); a group that represents the most important contributors to the total small pelagic fish catch^24,25^. This taxonomic group is known to display strong responses to environmental variability, especially fluctuations in phytoplankton availability^26^. These in turn are controlled by physical processes including vertical mixing and upwelling events related to monsoonal winds, as is the case for the larger WIO coastal region^9,27^. Due to the difficulty and cost of maintaining sustained *in-situ* observations in the region^28^, only a few studies have been achieved, reporting short-lived upwelling cells confined to the Tanzanian mainland coast that are suspected to be wind-driven^9^ or eddy-driven in the lee of the islands^29^. To-date, the identification of large-scale phytoplankton blooms and of mechanisms that could be responsible for their interannual fluctuations have not been investigated in detail. The previous work of Jury et al.^30^ evaluated the link between the total marine fish catch in East Africa, the WIO climate and coastal circulation at quasi-decadal and mean annual timescales using reanalysis data. In the present study, we focus on how the interannual changes in the alternating monsoons influence the chlorophyll (*Chl-a*, an index of phytoplankton biomass) availability. Understanding the mechanisms driving the interannual variability in *Chl-a* is important as this may directly influence on small pelagic fish stocks in Tanzanian waters.

Satellite observations, ocean model outputs, and in-situ measurements are used to examine biophysical processes likely driving the long-term variations in the catch of herrings, shads and anchovies in Tanzanian waters. We show that the yield of these small pelagics is synchronous with the available *Chl-a* concentrations on a year-to-year basis. The phytoplankton blooms are mainly influenced by the strength of surface currents during the Southeast monsoon, which result in “dynamic uplift” upwelling and westward advection of nutrients into Tanzanian waters. The interannual strengthening (weakening) of these mechanisms enhances (reduces) surface *Chl-a* concentration, owing to the interannual changes of monsoonal winds.

## Results

### Fisheries and *Chl-a* variations

**Figure 1:**
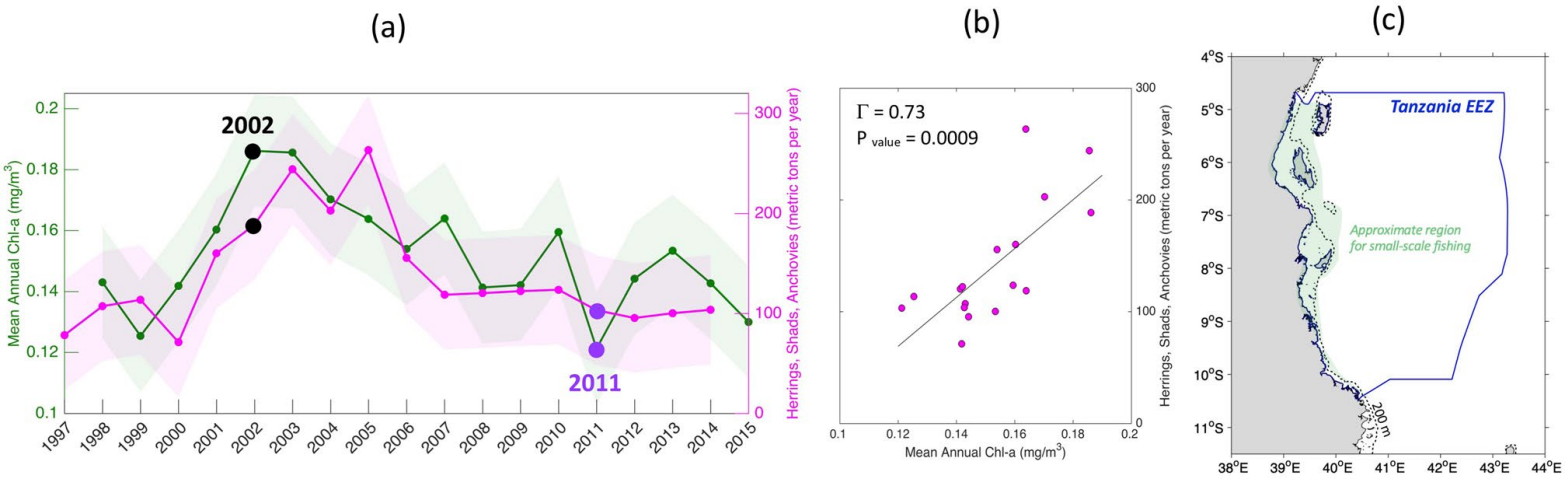
Interannual variability from 1997 to 2015 over the Tanzanian EEZ of herrings, shads, anchovies catch in metric tons and satellite Chl-a in mg/m^3^ (**a**) and their scatter plot (**b**). The light shadings on panel (**a**) represent ± 2 standard deviation for both variables. The approximate region for small-scale fishing within the Tanzanian EEZ is highlighted in light green on panel (**c**). The 200 m isobath derived from ETOPO2v1 global gridded database is represented by solid black line. The map on panel (**c**) was created by the authors using MATLAB software vR2015b (see https://uk.mathworks.com/products/new_products/release2015b.html and https://uk.mathworks.com/products/matlab.html).

For the period 1998–2014, the annual catch of herrings, shads and anchovies in the Tanzanian EEZ exhibited a clear increase when annual mean *Chl-a* concentrations were higher and a decrease when they were lower (Fig. 1, see “Methods” for details of the datasets used). The correlation between the recorded catches and the satellite-derived annual mean *Chl-a* concentrations is of 0.73 with a P_value_ of 0.0009 (Fig. 1b). This suggests, with the caveat that only 17 data points compose the timeseries, that the recorded catches parallel the corresponding satellite-derived annual mean *Chl-a* concentrations (Fig. 1b). Highest catches of small pelagics were recorded from 2001–2005, reaching about 200 metric tons in 2002, when the *Chl-a* is highest (Fig. 1a). Both annual mean *Chl*-a concentrations and catches increased prior to the plateauing of 2001–2005 and both declined thereafter (Fig. 1a). In 2011, the annual mean *Chl-a* concentration reached a historical minimum of 0.12 mg/m^3^, synchronously with a decline in the catch (Fig. 1a).

The significant positive relationship between small pelagic fish annual mean catch and *Chl-a* raises the question—what environmental factors drove the high *Chl-a* in 2002 and the increased harvest of small pelagic fish from 2001–2005 in the Tanzanian EEZ?

**Figure 2:**
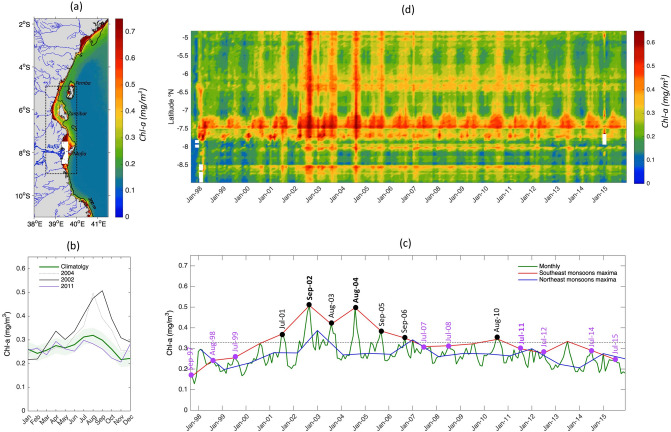
Satellite Chl-a seasonal and interannual variations over the Tanzanian coastal waters from 1997 to 2015. (**a**) Chl-a climatological annual mean in mg/m^3^. Data on the Rufiji river outflow area are masked in white. The 200 m isobath derived from ETOPO2v1 global gridded database is represented by solid black line. The thin blue lines on the grey land mask indicate the rivers positions. The major river (Rufiji) is highlighted with a thick blue line. The map on panel (**a**) was created by the authors using MATLAB software vR2015b (see https://uk.mathworks.com/products/new_products/release2015b.html and https://uk.mathworks.com/products/matlab.html). (**b**) The corresponding annual cycles averaged over the boxed region for the climatology, 2002, 2004 and 2011 are in green, black, grey and purple respectively. The light green shading represents ± 2 standard deviation from the climatology. (**c**) Monthly timeseries of Chl-a concentrations from September 1997 to December 2015 (in green). The Chl-a maxima of Southeast monsoons and Northeast monsoon (i.e. the maximum Chl-a attained during the season) are superimposed in red and blue respectively. The values falling on upper side of the 0.328 mg/m^3^ Chl-a level, which is the mean of the Chl-a maxima timeseries (dashed horizontal line), indicate high Chl-a years and are highlighted in black dots and those falling on the lower sider, represent low Chl-a years and are in a purple dots. (**d**) Chl-a meridional variations over the coastal area inside the black box (**a**) are presented by a time–space diagram.

Herrings, shads and anchovies prefer coastal habitats^31^. A spatial and temporal variability analysis of satellite Chl-a data was undertaken for the period 1997–2015 and for the shelf area indicated in Fig. 2a (see Supplementary Text 1 for more details on the choice of region). The spatial distribution of the climatological annual mean *Chl*-a concentration shown in Fig. 2a confirms the oligotrophic nature of these waters^18,21^, with concentrations rarely exceeding 0.25 mg/m^3^ over the majority of the area.

The green line (Fig. 2b) shows clear seasonal changes between the Northeast monsoon (i.e., from December to March) and the Southeast monsoon (i.e. from May to September). This seasonal variability is characterised by two annual maxima, with higher *Chl-a* during the Southeast monsoon (up to 0.32 mg/m^3^ from July to September) than the Northeast monsoon (up to 0.26 mg/m^3^ from January to February). Note that the *Chl-a* peak in April (inter-monsoon period) relates to increased riverine inputs and is discussed in the Methods section. At the interannual scale (Fig. 2c, green line), the Southeast monsoon *Chl-a* maxima (Fig. 2c, red line) are generally greater than the Northeast monsoon *Chl-a* maxima (Fig. 2c, blue line). The Southeast monsoon *Chl-a* maxima (Fig. 2c, red line) draws attention to September 2002 (Sep02 hereafter) as the month with the largest *Chl-a* concentration (0.6 mg/m^3^) and August 2004 (Aug04 hereafter) as the second highest *Chl-a* peak (0.56 mg/m^3^). Southeast monsoons with a relatively low *Chl-a* maximum (Fig. 2c, red line) are also observed as in 1998, 2011 and 2015 (purple dots). Here, we choose to focus on the example 2011 minimum (0.29 mg/m^3^, approximately half of the observed maximum) as it stands out in the annual *Chl-a* and fisheries timeseries in Fig. 1a and it can be considered a neutral year, unlike 1997–98 and 2015/16, which were super El-Niño and strong Indian Ocean Dipole (IOD) years^32^. Indeed, such decadal oscillations cause low *Chl-a* and warming over the WIO^33^. On the basis of Southeast monsoon *Chl-a* maxima during 1997–2015, all “highs” (Fig. 2c, black dots) and “lows” (Fig. 2c, purple dots) can be distinguished as the values falling on either side of the 0.33 mg/m^3^*Chl-a* level, which is the mean of the *Chl-a* maxima timeseries (dashed horizontal line). These composites of “highs” and “lows” are investigated below in “Results”, in addition to 2002 and 2011 examples.

Comparison of the *Chl-a* monthly means for 2002 and 2004 with the annual climatological cycle (Fig. 2b, black and grey lines) shows a near doubling of concentrations relative to the climatological values during September and August for these years. The *Chl-a* monthly mean for 2011 southeast monsoon months is also almost two standard deviations lower than typical conditions, with concentrations down to 0.26 mg/m^3^ (Fig. 2b, purple line).

The spatial and temporal extent of the 2002 and 2004 peaks and the 2011 decline can be further illustrated by the meridional variation of *Chl-a* over the Tanzanian coastal region (Fig. 2d). It shows that these extremes influence the whole Tanzanian coastline. Maximum *Chl-a* concentrations reach up to 0.46 mg/m^3^ (outside of the area between 7 and 7.6°S where it can exceed 0.65 mg/m^3^) every Southeast monsoon from 1998 to 2000 and 2006 to 2015. By contrast, the *Chl-a* level during the period between 2001 and 2005, is much higher, reaching 1 mg/m^3^ in Sep02 and Aug04.

### *Chl-a* response to monsoonal variability

**Figure 3:**
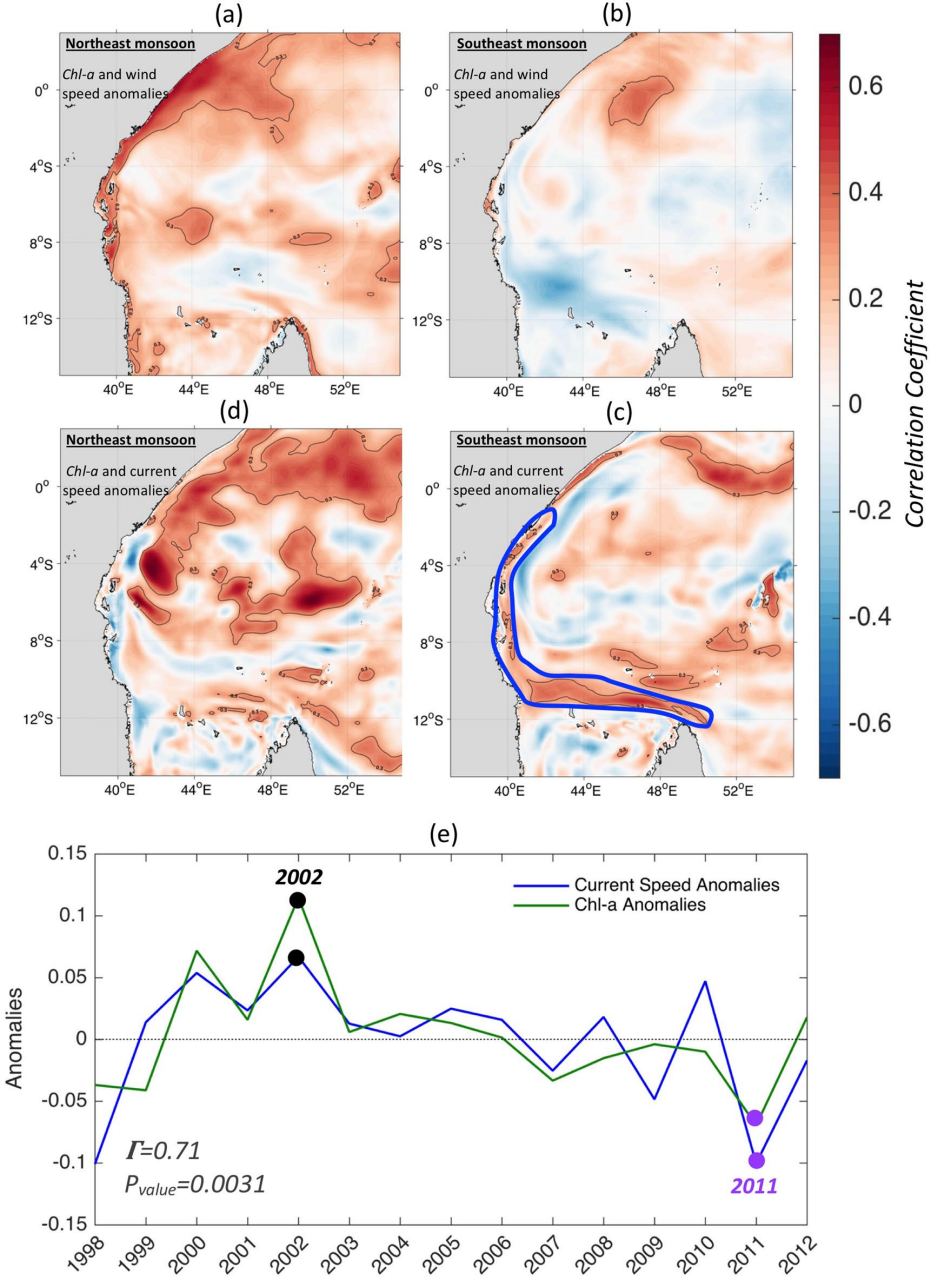
Impact of the change of monsoons on the Chl-a distribution along the East African coast during the period 1998–2012. Spatial correlation of surface Chl-a anomalies with wind (**a**), and surface currents anomalies (**d**) from the model level during the Northeast monsoons, and same for the Southeast monsoons (**b**,**c**) respectively. Note that the surface currents used are from the model first level (and correlation results remain similar for the first 100 m depth). Maps on panels (**a**–**d**) were created by the authors using MATLAB software vR2015b (see https://uk.mathworks.com/products/new_products/release2015b.html and https://uk.mathworks.com/products/matlab.html). (**e**) Timeseries of modelled surface currents and Chl-a anomalies during Southeast monsoons from 1998 to 2012 along the NEMC and EACC flows defined by their approximate positions as delimited by the thick blue contour on panel (**c**). 2002 peaks and 2011 drops in surface currents and Chl-a anomalies are highlighted in black and purple dots respectively.

To gain insight as to why the highest *Chl-a* was observed during 2002 and 2004, we considered whether both annual *Chl-a* maxima were caused by the same physical mechanism. For this we calculate a point by point correlation of surface *Chl-a* anomalies with wind speed and current speed anomalies over the WIO region from the high resolution (1/12°) global model NEMO, forced by reanalysis surface fluxes from 1958 to 2015. Note that the model is able to reproduce the key features of the surface circulation and associated monsoonal variability over our study area (see “Methods” for details). If similar mechanisms, related to wind or current speed, are causing the *Chl-a* changes in both seasons, one should expect the same sign of correlation along the East African coast. The aim is to see how the alternating monsoon affects the seasonal productivity. The correlations for the Northeast and Southeast monsoons over 1998–2012 are found to be in sharp contrast as shown on Fig. 3a–d. During the Northeast monsoons the modelled surface *Chl-a* and wind speed anomalies show high positive and significant (up to 0.7 with P_value_ < 0.05) correlations along the Tanzanian and Kenyan coasts (Fig. 3a), suggesting the occurrence of Ekman upwelling or enhanced vertical mixing. In contrast, no significant correlations were found during the Southeast monsoons (Fig. 3b), which excludes a wind driven upwelling mechanism for that season. Instead, surface *Chl-a* anomalies correlate (up to 0.4 with P_value_ < 0.05) with surface current speed anomalies near the Tanzanian and Kenyan coasts during the Southeast monsoons (Fig. 3c). Conversely, significant correlations between surface currents and *Chl-a* are absent in the Northeast monsoon months (Fig. 3d), except by the Somali Zanzibar Confluence Zone (SZCZ), at ~ 3.5°S where the Somali Current (SC) and EACC meet before deviating away from the coast, which initiates upwelling and enhanced primary production^34^.

Wind-induced upwelling and/or mixing likely cause the enhanced *Chl-a* during the Northeast monsoon along the East African coasts. Vertical mixing was considered as insubstantial in a previous analysis based on wind speed cube index^9^. However, wind-driven upwelling, a well-known upwelling process in coastal regions^35^, has been confirmed along the Tanzanian and Kenyan coasts during Northeasterly winds^9,27^.

What drives the seasonal *Chl-a* response to the Southeast monsoon along the Tanzanian and Kenyan coastlines acts independently from the local wind forcing and is rather related to surface currents. Such a response indicates the occurrence of two possible mechanisms, a dynamic uplift upwelling or increased advection of nutrients into the region. Dynamic uplift upwelling acts through an intensification of the along-shelf current causing a shallower thermocline and upwelled waters^36^. For the Tanzanian/Kenyan coastal area, the EACC is the obvious candidate for this scenario as during the Southeast monsoon the modelled current velocity intensifies more than twofold relative to speeds during the Northeast monsoon (from 0.8 to > 2 m/s; Supplementary Figs S2e and S3e). Furthermore, the sudden rise in the topography of the Tanzanian/Kenyan coastal transition zone, due to the archipelago of islands, seamounts and narrow channels, should favour more across-slope motion^37^ and hence a stronger phytoplankton response to upwelled nutrients than in open ocean areas^38^.

More synoptically, the area of high correlation between *Chl-a* and the surface current speed extends much farther east of the African shelf along 10°S to the northern tip of Madagascar, generally in line with the path of NEMC. This then raises the question of whether the horizontal advection of nutrients along this pathway works as an additional mechanism contributing to elevated levels of *Chl-a* along the Tanzanian and Kenyan shelf.

### Dynamic uplift as a key driver of the Southeast monsoon phytoplankton bloom

To better understand the mechanisms behind the *Chl-a* response during the Southeast monsoons, we next assess the occurrence of the dynamic uplift upwelling and horizontal advection by focusing on the intense blooms of Sep02 and Aug04. The characteristics of Sep02 bloom are presented below and those of Aug04 in Supplementary Fig. S4 and Text 2.

**Figure 4:**
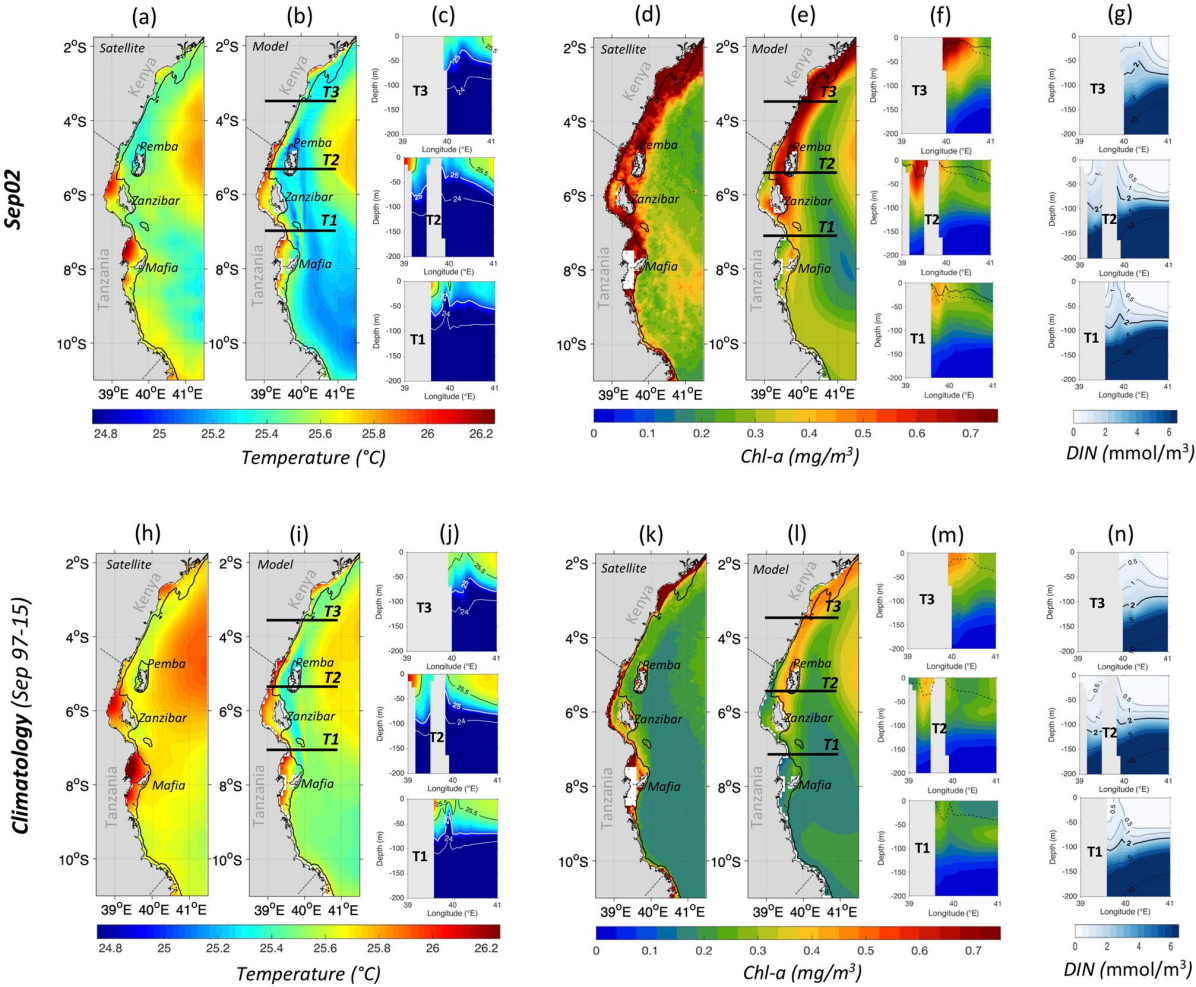
Surface and subsurface signatures of dynamic uplift upwelling along the Tanzanian and Kenyan coasts during Sep02 relatively to the climatology (1997–2015). SST in °C from satellite data (**a**,**h)** and the model (**b**,**i**) and surface Chl-a in mg/m^3^ from satellite data (**d**,**k**) and the model (**e**,**l**). Satellite Chl-a data on the Rufiji river outflow area are masked in white on panels (**d**) and (**k**). The 200 m isobath derived from ETOPO2v1 global gridded database are represented by solid, dashed and dotted black lines respectively. Cross-sections (T1 to T3) of modelled temperature in °C (**c**,**j**), Chl-a in mg/m^3^ (**f**,**m**) and DIN in mmol/m^3^ (**g**,**n**) are displayed along three locations as indicated on panels (**b**,**e**,**i**,**l)** with back horizontal lines. The MLD in m of Sep02 and the climatology are represented by the black solid and dashed lines respectively on panel (**f**). The 2 mmol/m^3^ isopleth is highlighted with a thick black line on panels (**g**) and (**n**). Maps on panels (**a**,**b**,**d**,**e**,**h**,**i**,**k**,**l**) were created by the authors using MATLAB software vR2015b (see https://uk.mathworks.com/products/new_products/release2015b.html and https://uk.mathworks.com/products/matlab.html).

The upwelling signature in Sep02 is first examined in the Sea Surface Temperature (SST) and surface *Chl*-a relative to the 19-year climatological mean (1997–2015) as derived from satellite data and the model (Fig. 4). The satellite data reveal a coastal band of elevated *Chl-a* (up to 1 mg/m^3^) from Mafia Island (~ 8.5°S) to Kenya (~ 2°S), contrasting with deeper waters further East with low *Chl-a* (down to 0.15 mg/m^3^) (Fig. 4d,k). *Chl-a* concentrations intensified in Sep02 to about three times their climatological values (Fig. 4d,k). In most cases, the distributions of high *Chl-a* in the model match well with those in satellite observations (Fig. 4e, l, d, and k), with just few exceptions (see “Methods” for model/satellite *Chl-a* inconsistencies). The observed SST along the coastal band (between Mafia and Zanzibar islands, in the Pemba Chanel and further north towards Kenya) has cooled down to 25.2 °C compared with SST of 25.7 °C in the climatology (Fig. 4a,h). The modelled SST for the same coastal band also shows low values, decreasing from 25.3 °C in normal conditions to below 24.9 °C over a similar spatial extent to satellite observations (Fig. 4b,i). Overall, the clear pattern of high *Chl-a* concentrations associated with cooler waters along the coastline during Sep02 and in the climatology is suggestive of either intensified upwelling or enhanced vertical mixing.

Analysing the modelled MLD across three vertical cross-sections along the elevated *Chl-a* coastal band between Mafia and Northern Kenya (T1–T3, see Fig. 4e for exact positions) reveals a shallower MLD during Sep02 (solid lines, Fig. 4f) than the climatology (dashed lines, Fig. 4f). This suggests that the elevated *Chl-a* in Sep02 is not due to the intensification of upper ocean mixing but rather coastal upwelling which shoals the mixed layer.

The upwelling subsurface signature can be assessed in the modelled temperature, *Chl-a*, and Dissolved Inorganic Nitrogen (DIN) along sections T1–T3. In the climatological Southeast monsoon, sections T1–T3 indicate cool SST, high *Chl-a* and elevated nutrients near the coast (Fig. 4j,m,n), typical of an upwelling regime. The 25 °C isotherm rises at around 40 °E from deep layers (< 80 m) to the near surface (30–50 m). This results in an uplift of the 25.5 °C isotherms on sections T2–T3 in the climatological September. These cooler temperatures are accompanied by high *Chl-a* concentrations of 0.4–0.5 mg/m^3^ in the upper 80 m. The elevated *Chl-a* is also consistent with the doming of DIN isopleths on sections T1 to T3 near the coasts. During Sep02, the same situation occurs but with much more intensity (Fig. 4c,f,g). The cold waters with elevated nutrients are closer to the surface than in the climatology, leading to higher *Chl-a* concentrations (Fig. 4f,m). The uplift of the 25 °C isotherm in Sep02 reaches 30–40 m on section T3 and 20 m (30 m) on the rest of sections, while residing around 50 m and 30 m respectively in the climatology (Fig. 4c). The elevated *Chl-a* over 80 m depth exceeds 0.75 mg/m^3^ in Sep02, which is almost the double of the climatological values (Fig. 4f). The prominent peak in *Chl-a* near the coasts coincides with a more accentuated doming of the 2 mmol/m^3^ DIN isopleth for Sep02 than in the climatology (Fig. 4g).

Kenyan and Tanzanian waters generally have few in-situ biogeochemical observations. Fortuitously however, an in-situ cruise dataset (see “Methods” for details) of several biophysical variables exists for Aug04, a period when one of the pronounced *Chl-a* maxima occurred. These data allow validation of the model performance during an extreme Southeast monsoon and confirms the presence of upwelling (Supplementary Fig. S5 and Text 3).

The investigation of upwelling occurrence can be expended from the extremes like 2002 to composites of all Southeast monsoons with “high” and “low” *Chl-a* as defined from Fig. 2c (black and purple dots), using the satellite and model data. The composites of all *Chl-a* “highs” show that the upwelling is pronounced with elevated DIN and colder temperatures in the surface layers during those events (Supplementary Fig. S6a–g). In contrast, the composite of all *Chl-a* “lows”, illustrates a reduced upwelling signal (Supplementary Fig. S6h–n). In summary, our observational and modelling results identify that upwelling along the coasts of Tanzania and Kenya sustain the delivery of cold and nutrient-rich waters to surface layers during the Southeast monsoon with an intensity that varies interannually.

### Role of horizontal advection in the Southeast monsoon phytoplankton bloom

**Figure 5:**
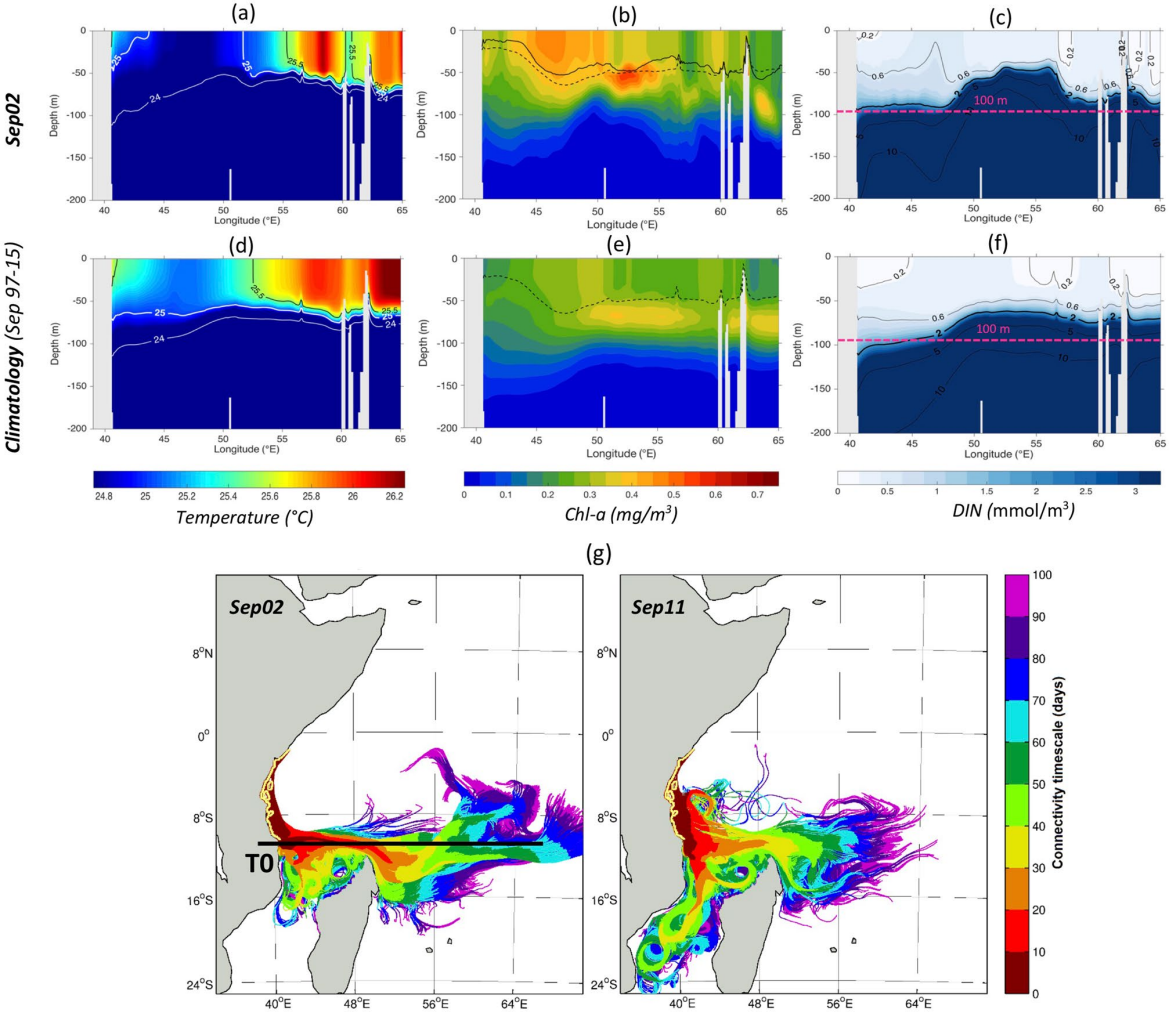
An Advective impact along the NEMC path of nutrients rich and cold waters from the Madagascar northern tip to the Tanzanian coast during Sep02 and the climatology (1997–2015). A cross-section (T0) from 39 to 65°E of modelled temperature in °C (**a**,**d**), Chl-a in mg/m^3^ (**b**,**e**) and DIN in mmol/m^3^ (**c,f**) is displayed along the latitude line 10° S as indicated with the black line on panel (**g**). The MLD in m of Sep02 and the climatology are represented by the black solid and dashed lines respectively on panel (**b**). The 2 mmol/m^3^ isopleth is highlighted with a thick black line on panels (**c**) and (**f**). (**g**) Trajectories of virtual particles backtracked from the East African coastal zones (marked with light yellow dots) in Sep02 and Sep11, back to their upstream sources in the surface Indian Ocean up to 100 days prior. Colours denote the ‘connectivity timescale’—i.e. the minimum amount of time, in days, required for waters from a given area to reach any of the end points on the East African coast. Note that reds/oranges paths are plotted on top of blues/purples to highlight the most rapid advective pathways in both cases. Maps on panel (g) was created by the authors using MATLAB software vR2013a (see https://uk.mathworks.com/videos/r2013a-release-highlights-75269.html and https://uk.mathworks.com/products/matlab.html).

In addition to dynamic uplift upwelling, a vertical cross-section in the modelled temperature, *Chl-a* and DIN along latitude 10.5° S, from 39° to 73° E (section T0, Fig. 5), illustrates a potential advective impact along the NEMC path (40°–50° E). The T0 transect passes through areas of enhanced mixing and nutrient-rich waters during the Southeast monsoon, located around the northern tip of Madagascar, in the vicinity of Aldabra and Cosmoledo islands and around the Mascarene plateau (Supplementary Fig. S7). This is also where high nutrients and *Chl-a* were observed from *in-situ* data in the Southeast monsoons of 2001 and 2002^39^. In our case study, the modelled climatological September reveals temperatures lower than 25.5 °C extending to the Tanzanian coast (41° E) from the tip of Madagascar (51° E) (Fig. 5d). The DIN isolines steepen near the Madagascar tip (51° E) and around the Mascarene plateau (55°–65° E), and nutrient-rich waters cover the NEMC advective pathway (40°–50° E) (Fig. 5f). Consequently, elevated *Chl-a* values ranging between 0.35 and 0.4 mg/m^3^ are found along that same path with a subsurface *Chl-a* maximum (0.45–0.5 mg/m^3^) located at 60–65 m near the Madagascar tip (51°E) and around the Mascarene plateau (55°–65° E) (Fig. 5e). During Sep02, the waters along the NEMC path are colder (< 24.5 °C, Fig. 5a) with higher *Chl-a* concentrations (> 0.6 mg/m^3^ with a subsurface maximum of 0.7 mg/m^3^ at 60-75 m, Fig. 5b) and enhanced doming of DIN isopleths (Fig. 5c) relative to the climatology. Note that similar patterns to Sep02 are found during Aug04, another intense *Chl-a* monsoon (see Supplementary Fig. S8). The interannual changes in *Chl-a*, DIN and temperature described along the NEMC path suggest that advection by the surface circulation may play a key role in those changes.

**Figure 6:**
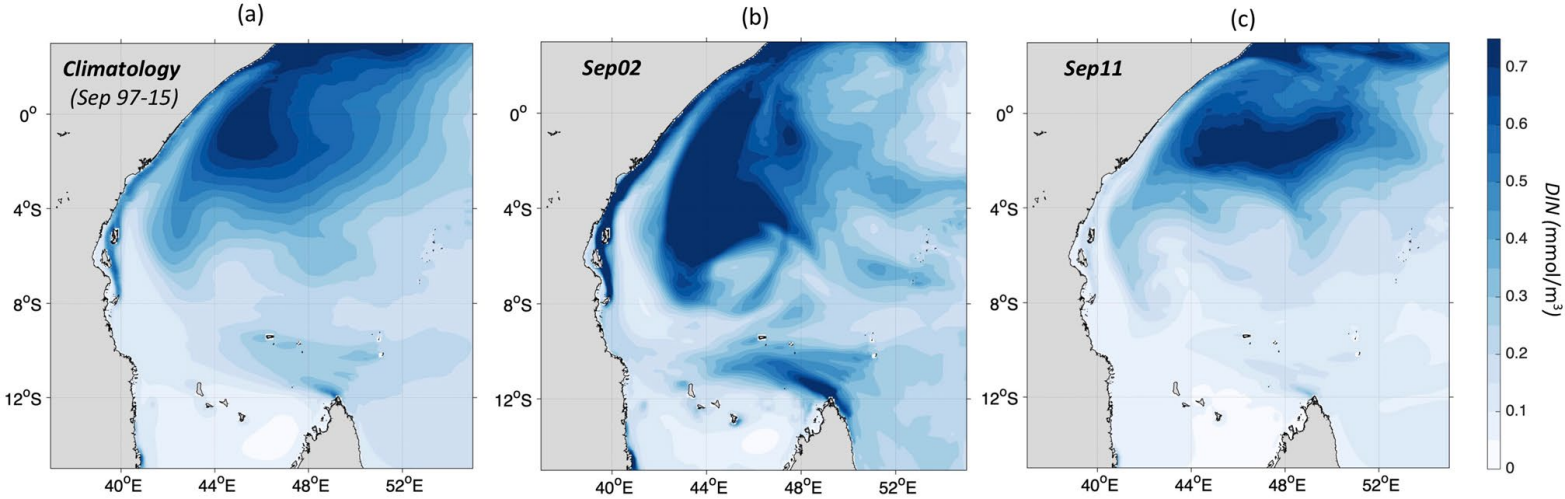
Nutrient-rich waters over the southern WIO during Sep02 and Sep11 relatively to the climatology (1997–2015). Modelled Surface DIN in mmol/m^3^ in (**a**) the climatology (1997–2015), (**b**) Sep02 and (**c**) Sep 11. Maps on panels (**a**–**c**) were created by the authors using MATLAB software vR2015b (see https://uk.mathworks.com/products/new_products/release2015b.html and https://uk.mathworks.com/products/matlab.html).

To further investigate the role of advection in transporting nutrient rich waters to the East African coast, two Lagrangian particle back-tracking experiments forced by model currents using the ARIANE analysis tool^40^ were performed (see “Methods” for details). Note that due to the complexity of the flow and its mesoscale nature, advective time scales cannot be easily derived from an Eulerian velocity field, hence the use of Lagrangian particle tracking here. Virtual particles were uniformly distributed in the model grid, with 9 particles per cell (~ 1 particle per 10 km^2^) along Tanzanian and Kenyan shorelines spanning up to 15 km from the coast. These particles are tracked backwards to find their sources 100 days upstream of their final positions, the East African coastline. The design of these two experiments is analogous to that in Popova et al.^41^. Each experiment releases up to a total of 3,735 particles at the ocean surface, but one with arrival points in Sep02 (example of a strong bloom) and the other in September 2011 (Sep11 hereafter, contrasting example of no-bloom). Figure 5g shows the particles trajectories and Supplementary Fig. S9 their densities. In 2002, a substantial pathway comes from areas around the northern Madagascar tip while in 2011 it originates from the Mozambique channel. It is apparent that direct connectivity via the NEMC between Madagascar’s northern tip and the Kenyan/Tanzanian coastline is markedly faster in Sep02 (10–20 days) compared to Sep11 (30–40 days). Trajectories densities along the NEMC from the northern tip of Madagascar to the East African coast are higher in Sep02 compared to Sep11 (Supplementary Fig. S9). Thus, we expect an increased transport of nutrients from a nutrient rich area such as that found at the northern tip of Madagascar in 2002^39^. This is confirmed by examining cross-sections of modelled DIN perpendicular to the NEMC flow at several locations (49° E; 47° E; 45° E; 43° E and 41° E) from the northern tip of Madagascar up to the Tanzanian coast (Supplementary Fig. S10). These show that the core of the flow has increased DIN (isopleths closer to the surface) from section to section towards the Tanzanian coast in Sep02, relatively to Sep11 and the climatology. Additionally, maps of surface DIN over the WIO for 2002, 2011 and the climatology (Fig. 6) show enhanced (reduced) surface DIN at the tip of Madagascar in 2002 (2011) relative to climatology, providing a source of nutrients for the NEMC to advect. Note that this result can be generalized to composites of surface DIN during the *Chl-a* “highs” and “lows” (Supplementary Fig. S11), as determined from Fig. 2c (see details above in “Results”). In conclusion, the increase in trajectory densities and the higher nutrients across the path of the NEMC suggest an important advective role of the currents in bringing more nutrients to coastal East Africa from Madagascar’s northern tip in 2002 than in 2011.

### *Chl-a* response to interannual changes in the Southeast monsoon

It has been shown that current-induced upwelling (via the EACC) and advection (via the NEMC and EACC) are the main controls on *Chl-a* variability during the Southeast monsoon. In the following we focus on NEMC and EACC dynamics to address what differed during the Southeast monsoon with extreme *Chl-a* concentrations along Tanzanian/Kenyan coasts, such as 2002 with its intense biological response, and 2011 with its low *Chl-a* (cf. Figs. 1a, 2b,c). Furthermore, examining composites maps of different environmental fields like SST, *Chl-a* and surface currents of high minus low catch years, revealed similar conclusions (see Supplementary Figs. S12 and S13 and Text 4).

To verify whether an anomalous increase in current speed might explain the extreme *Chl-a* peaks and troughs, the interannual variations in surface currents and *Chl-a* anomalies along the NEMC and EACC were examined. The paths of the EACC and NEMC approximate position can be defined from the climatological position of the modelled currents (Supplementary Fig. S3e) as delimited by the blue line on Fig. 3c. The temporal variations of total mean current speed and *Chl-a* over the EACC and NEMC path (i.e. averaged in the blue area) during the Southeast monsoon (Fig. 3e) show strong and positive correlation of 0.71 (P_value_ = 0.0031) over the period 1998–2012 (see “Methods” for details on the 2012 limit). The year-to-year variability shows elevated (low) *Chl-a* concentrations paralleling a stronger (weaker) EACC/NEMC (Fig. 3e). There is a persistent increase in *Chl-a* and currents anomalies from 1998 to 2002, with a peak in 2002 (increase by 0.07 m/s and 0.12 mg/m^3^) followed by a persistent decrease from 2003 to 2012 with the absolute minimum reached in 2011 (decrease by − 0.1 m/s and − 0.07 mg/m^3^) (Fig. 3e).

This year-to-year *Chl-a*—currents relationship motivates further investigation into the cause of the anomalous increase (decrease) of the EACC and NEMC during some Southeast monsoons. To do this, we examine the regional wind stress curl for the examples of extreme peak and drop of *Chl-a* (2002 and 2011) and composites for all years of *Chl-a* “highs” and “lows” as determined from Fig. 2c. The aim here is to assess if the wind stress curl changes, which can set up changes in surface circulation, are concurrent with the surface current changes between high and low *Chl-a* years as in 2002 and 2011. However, note that other factors like Rossby waves generated also by wind stress curl forcing in the South Indian Ocean may remotely play a role in the variability of the NEMC and the SEC^42^.

**Figure 7:**
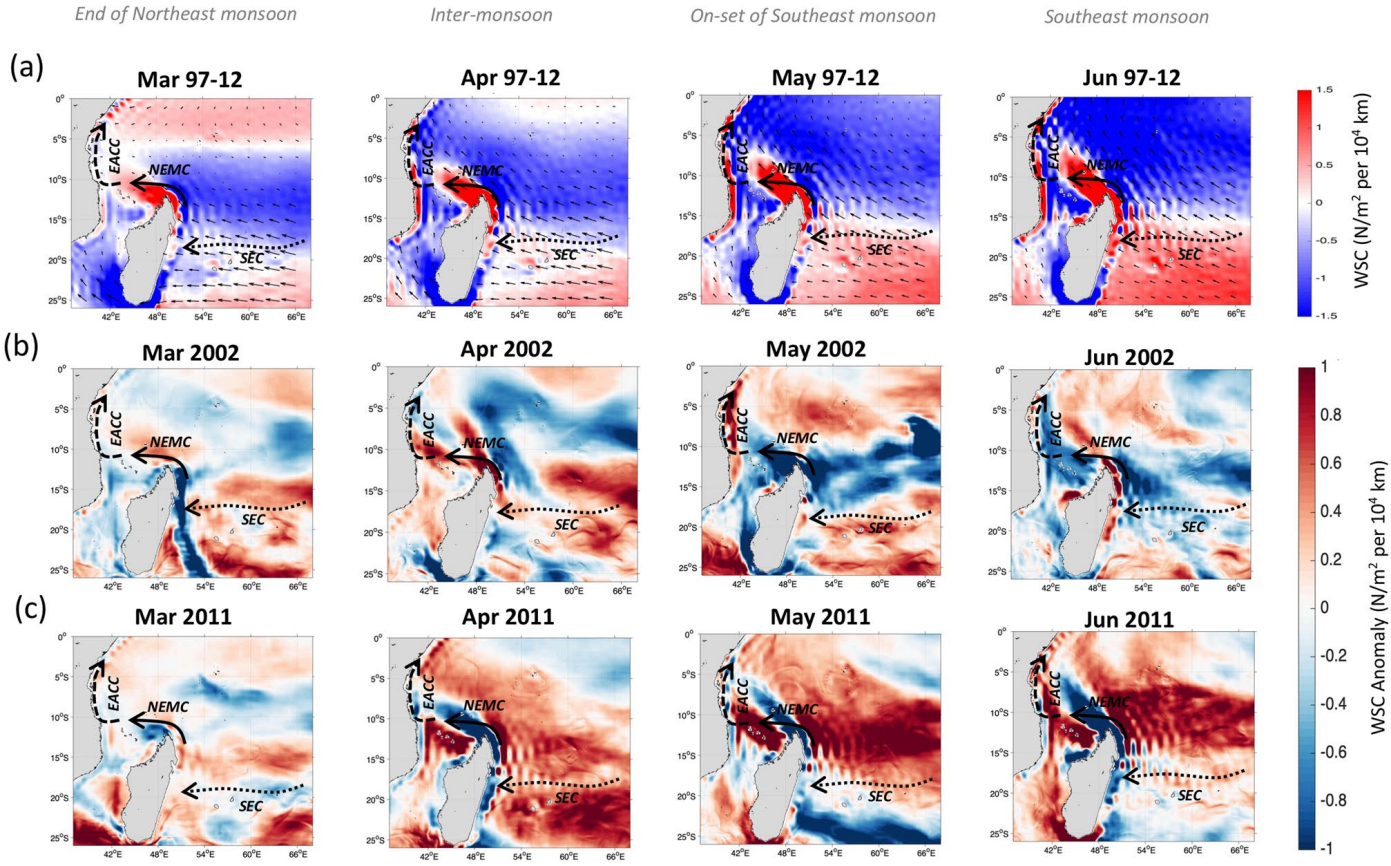
Wind Stress Curl (WSC) forcing over the southern WIO from March to June in (**a**) the climatology (1997–2012), (**b**) 2002, and (**c**) 2011. Wind stress vectors in (**a**) are displayed every 25 grid points. WSC anomalies from March to June (**b**) 2002 and (**c**) 2011, relatively to the period 1997–2015. A schematic view of the South Equatorial Current (SEC), North East Madagascar Current (NEMC) and East African Coastal Current (EACC) based on Schott et al.^20^ is superimposed to show the areas of influence of Southeast winds. Maps on panels (**a**–**c**) were created by the authors using MATLAB software vR2015b (see https://uk.mathworks.com/products/new_products/release2015b.html and https://uk.mathworks.com/products/matlab.html).

The wind stress curl fields over the WIO, compared with the climatology, prior to (March–April) and/or during the onset of (May–June) the Southeast monsoons with extreme *Chl-a* along the Tanzanian/Kenyan coasts are shown on Fig. 7. The climatological wind stress curl fields show predominantly strong positive values, ranging between 0.7 to greater than 1.5 N/m^2^ per 10^4^ km from March to June along the NEMC path and from May to June along the EACC path (Fig. 7a), while weaker positive anomalies of 0.3–0.7 N/m^2^ per 10^4^ km are detected along the SEC path from March to June.

Prior to the on-set of the Southeast monsoon of 2002 (March to April), the SEC path experiences wind stress curl anomalies of about 0.5–0.6 N/m^2^ per 10^4^ km (Fig. 7b). During those same months and in June, the wind stress curl over the NEMC path intensified by 0.6–1 N/m^2^ per 10^4^ km (Fig. 7b). Over the EACC path, the increase exceeds 1 N/m^2^ per 10^4^ km in April and May (Fig. 7b). Similar wind stress curl patterns are also seen from March to June 2004 (Supplementary Fig. S14), the other year with a high *Chl-a* response during the Southeast monsoon (Fig. 2). Conversely, the wind stress curl patterns in 2011 are almost a mirror image those of 2002 (Fig. 7c), being markedly weaker (by − 1 N/m^2^ per 10^4^ km) along the NEMC and EACC from March to June and moderately reduced (by − 0.4 N/m^2^ per 10^4^ km) along the SEC in March (although the local wind stress curl over the SEC path shows a marked intensification during April and May 2011). This coincides with the overall decrease of the NEMC and EACC current speed and subsequent reduction in *Chl-a* along coastal East Africa during the Southeast monsoon of 2011 (see Fig. 3e).

Similarly, the wind stress curl anomalies are analysed over the composites during March to June of all low and highs Chl-a years (Supplementary Fig. S15) as determined from Fig. 2c. The composites of all “highs” show an intensification of the wind stress curl exceeding 0.15 N/m^2^ per 10^4^ km and those of all “lows” a decrease of − 0.15 N/m^2^ per 10^4^ km over the path of the current of interest, in particular along the NEMC and the EACC paths (the decrease/increase of wind stress curl over the SEC path occurs during Marchs or Aprils). This result supports the idea that there are typical changes in wind stress curl over the South tropical Indian Ocean during high and low Chl-a Southeast monsoons for all highs versus all low years.

The interannual variations in wind stress curl over WIO, here related to the Southeast monsoon winds, will lead to changes in the currents. These in turn strongly influence the chlorophyll variability along the East African coasts. In particular, in “high” years, like 2002, the currents induce increased upwelling at the coast and the westwards advection of nutrient rich waters towards the coast, so enhancing productivity there.

## Discussion

The new results presented in this study highlight the response of small pelagic fish yield to *Chl-a* variability in East African waters and identify, for the first time, the large-scale physical mechanisms that control it.

The year-to-year catch of herrings, shads and anchovies (*Clupeiformes* and *Engraulidae*) appears to significantly correlate to the variations in annual mean *Chl-a* concentrations (cf. Fig. 1a). Extending the length of the timeseries could facilitate gain further confidence in the statistical significance of the *Chl-a* and fisheries relationship. However, this is limited by the availability of the fisheries record which ends in 2014. Although statistical significance does not necessarily mean ecological causality, we demonstrate the physical mechanisms behind the *Chl-a* and fisheries interannual variations. The *Chl-a* and catch relationship can also be explained partly by the characteristics and behaviour of these species. They are small in size (< 27 cm, see “Methods”) and mainly planktivorous^43^. Most adult Clupeiformes increase in abundance^44^ when food availability increases (i.e. higher *Chl-a*). Although the lack of monthly fish catch data prevents a direct linkage to the seasonal phytoplankton bloom timing, studies of *Clupeiformes* in other coastal regions have shown that most species exhibit a seasonal reproductive period^45^. While the herrings and shads reproduce throughout the year in tropical regions^46^, the regional anchovy species, which are found along the Tanzanian coast^47^ spawn near the surface between October and March^48^. Since the survival of larval stages is dependent upon the variable ocean conditions^49,50^, most small pelagic fish spawn in locations and during seasons that minimize these losses^9^. In our case study, the strong surface currents (and moderate mixing, Supplementary Fig. S7) that promote higher *Chl-a* occur between May and September, are out of synch with the period of spawning and breeding of anchovies from October to March. Interannually, the simultaneous reduction in catches, *Chl-a* and currents, after the period 2001–2005, which continued until 2014, suggests a strong environmental influence rather than changes in fishing efficiency. Furthermore, Jury et al.^30^ reported that environmental factors in the Tanzanian and Kenyan coastal zones play a significant role in the fluctuations of the overall fisheries catch. They suggested that the SEC regulates some of these changes^30^. In our study, the underlying large-scale physical mechanisms affecting *Chl-a* in East Africa in both monsoons and interannually, which in turn influence the fluctuations of the small fish catch, have been identified and discussed.

Our work shows that the variability of *Chl-a* in the study area is determined by upwelling and advective processes supplying nutrients. Ekman upwelling due to the Northeasterly winds drives interannual variability in the Northeast monsoon. Current-induced upwelling takes place combined with advection of nutrients caused the interannual variability in the Southeast monsoon. The latter mechanisms drive a stronger biological response than during the Northeast monsoon. The interannual variability shows that *Chl-a* is higher (lower) when surface currents accelerate (weaken) significantly, allowing more nutrient rich waters to be upwelled to the surface and/or advected from further Southeast. The strength of these surface currents is influenced by large-scale changing monsoon winds. Though monsoon winds are known to promote phytoplankton growth in the Indian Ocean^51^ via Ekman driven upwelling^52^, the novelty here is that monsoonal winds through their impact on currents drive a less common type of upwelling, a dynamic uplift and a westward advection of nutrient-rich waters. The resultant increase (decline) in *Chl-a* leads to higher (lower) small pelagic fish catch (Fig. 1a). Such changes in the food supply to lower trophic level species are highly likely to impact the sustenance and economy of East African coastal populations that depend on marine resources^1^.

While anomalous variations in the large-scale monsoon patterns during 2002 have benefited the economy of coastal East Africa with increased *Chl-a* and fish yield, they resulted in opposite effects in other parts of the Indian Ocean as the Indian subcontinent with a catastrophic drought and economic losses of billions of dollars^53^. We have also shown how a decline in the *Chl-a* may occur along the Tanzanian coast during a weak Southeast monsoon scenario (such as 2011). Other periods of low biological response occurred during the super El-Niño and strong IOD years of 1997–1998 and 2015/16 (Fig. 2d). Furthermore, Currie et al.^33^ found a strong negative *Chl-a* anomaly during El-Niño and positive IOD of 1997–1998 over the WIO and particularly the East African coast. This difference in the oceanic biological response to El Niño/Southern Oscillation (ENSO) (1997–1998 and 2015–2016) and to anomalous monsoon years (2002, 2004 and 2011), suggests that interannual variations in the monsoon may become the main control for the regional response when the ENSO mode is off or weak.

Further investigation is required to fully elucidate the interactions between the different seasonal and interannual variability modes (Monsoon circulation, ENSO and IOD) and coastal East Africa. The occurrence of coupled feedback can have considerable implications on regional WIO climate variability^54^, which would be of broader importance to the Tanzanian economic sector as it is among the most vulnerable to climate change-driven impacts on fisheries^15^. Improving our understanding of the physical controls on phytoplankton blooms in this region will contribute to better projections of changes in productivity and fisheries in response to a changing climate. Continuous monitoring of both oceanic and atmospheric conditions is required but relying on cost-effective tools like remote sensing and numerical modelling could be the key to overcoming the lack of *in-situ* observations in the WIO, and along the East African coast in particular. Multi-model simulations performed in the framework of the *IPCC AR5* project a long-term decline of primary production in the tropics following temperature-driven stabilisation of ocean stratification under the RCP8.5 “business as usual scenario”^55^. However, in the background of this large-scale general decline of ocean productivity, a strengthening and lengthening of the Southeast monsoon over the Indian Ocean is projected by the *IPCC AR5*^3^ which could favour increased frequency of strong and wide-spread phytoplankton blooms leading in turn to increased fish stock along coastal East Africa. A combination of continuous monitoring through remote sensing and projected monsoon changes from climate models, could hence provide important information for policy and resource management decision-making.

## Methods

### Satellite and in-situ observations

Monthly satellite-derived *Chl-a* concentrations were acquired from the Ocean-Colour Climate-Change Initiative (OC-CCI) project (https://www.esa-oceancolour-cci.org/), at a spatial resolution of 4 km. This product is the most consistent timeseries of multi-satellite (MODIS-Aqua, SeaWiFS and MERIS) global ocean colour data^56^. Its monthly composites cover September 1997 to June 2018 but the period 1997–2015 is used here to derive the climatological mean in order to be consistent with the model data (see below).

It has been argued that bio-optical algorithms, including the OC-CCI processing might overestimate satellite *Chl-a* concentrations in coastal and/or very shallow waters^57^. The main reason is that these optically complex waters can be impacted by the presence of other optical constituents such as suspended sediments, particulate matter and/or dissolved organic matter^60^. More precisely, the underwater reflectance in shallow areas generates high water-leaving radiance in the near-infrared wavelengths which in turn would overestimate the correction term. However, suspended material in areas with abundant coral reefs are considered low which is the case of the west coasts of Zanzibar and Pemba islands and along the majority of the Tanzanian coast^59–61^. The only exception is the Rufiji river outflow area, west of Mafia island (see Fig. 2a), which is the major river along the Tanzanian coast and where suspended material is significant^62^. In addition, mainland coastline of Tanzanian has several riverine inputs as illustrated on Fig. 2a, which can explain the enhanced *Chl-a* values exceeding 0.7 mg/m^3^. This bias influences the seasonal cycle of *Chl-a* resulting in the April peak (climatological, 2002 and 2004) (Fig. 2b), which is in synch with the “long” rains period ^63^, a proxy for river discharges. For the rest of the results with remotely sensed *Chl-a*, the focus is mainly on the coastal areas which comprise mainly Case I waters.

The reprocessed Met Office *Operational-Sea-Surface-Temperature-and-Sea-Ice-Analysis* (OSTIA) SST product was also acquired. This multi-satellite and global dataset is made available by the Copernicus Marine Environment Monitoring Service (CMEMS) (https://marine.copernicus.eu/services-portfolio/access-to-products/) from 1985 to 2018. The SST data are provided daily at a spatial resolution of 0.05°. We compute monthly means during the study period 1997–2015 to match the observed and modelled *Chl-a* dataset.

Another satellite product exploited in this study is the altimetry derived absolute geostrophic currents processed by AVISO (Archiving, Validation and Interpretation of Satellite Oceanographic Data) and distributed by CMEMS (https://marine.copernicus.eu/services-portfolio/access-to-products/). These are the daily geostrophic zonal and meridional velocities gridded at 25 km spatial resolution from the delayed time “Update” DUACS-DT2018 version. We compute the climatological monthly means over the period 1997–2015 for comparisons purposes with the modelled surface currents.

Finally, in-situ data obtained during the African Coelacanth Ecosystem Programme (ACEP) are used to validate model subsurface features over the study area. Specifically, we use the CTD measurements (temperature, fluorescence) and nutrient data (nitrate/nitrite) collected on an oceanographic cruise by the R/V Algoa in Tanzanian waters during August 2004. More information about the sampling details of the oceanographic cruise can be found in Scott^66^, ACEP^67^ or the CSIR geoportal (https://geoportal.csir.co.za/saeon_/metadata/custodian.2011-01-04.8036881558/custodian.2011-01-04.8036881558-MetadataCollection/meta129415038881). Although the in-situ dataset is subject to uncertainties arising from the measurements and sampling accuracy, it shows good agreement with the independent model results. The fact that they both show similar results strengthens the trust in each of those datasets (see Supplementary Figs. S5, S4 and Supplementary Text 3).

### Model overview, data and validation

The modelled surface and vertical sections of temperature, surface currents, winds and MLD are obtained from version 3.6 of the global ocean model NEMO (Nucleus for European Modelling of the Ocean)^68^ spanning the period 1958–2015 with outputs stored as 5-day means. The model has a horizontal spatial resolution of 1/12° and 75 vertical levels with finer grid spacing near the sea surface. The model was forced with reanalysis atmospheric data from the Drakkar Surface Forcing dataset version 5.2, which supplies 2 m air temperature, 2 m humidity, 10 m winds, surface radiative fluxes and precipitation^69,70^. The bottom topography is represented as partial steps and bathymetry is derived from ETOPO2V2.

A plankton ecosystem model, MEDUSA-2 (Model of Ecosystem Dynamics, nutrient Utilisation, Sequestration and Acidification), is coupled to NEMO over the period 1990–2015 to represent the biogeochemistry (for details see Yool et al.^71^). Note that we utilize in this study only the period of September 1997 to December 2015 for the selected physical (temperature, surface currents, MLD, wind stress) and biochemical (*Chl-a*, DIN) modelled outputs, except for the wind that is used until December 2012. The MLD in the model is calculated as the depth at which the temperature is at least 0.1 °C different from the surface temperature.

To calculate wind stress curl (WSC), the wind stress fields from the model (i.e. the reanalysis dataset used for model forcing) are used in the following equation:

$$WSC = \frac{{\partial \tau_{y} }}{\partial x} - \frac{{\partial \tau_{x} }}{\partial y}$$

where $\tau_{x}$ and $\tau_{y}$ are the zonal and meridional components of the wind stress and x and y are the zonal and meridional dimensions.

NEMO and MEDUSA-2 have been successfully used to study ocean dynamics in the Indian Ocean^72^. Comparisons with satellite gridded data show that the modelled output accurately reproduces the main surface circulation features at the appropriate seasonal scale over the central WIO (i.e. − 15° to 12° N and 37° to 73° E) as presented in Supplementary Figs. S2 and S3. Here, we consider the 1997–2015 climatological February (August) representative of the Northeast (Southeast) monsoon. During the Northeast monsoon, the model and satellite *Chl-a* spatial patterns show an overall good agreement despite the generally larger simulated values (by ~ 0.2 mg/m^3^). The highest *Chl-a* values (> 0.6 mg/m^3^ in the model and > 0.4 mg/m^3^ in observations) are depicted north of the South Equatorial Counter Current (SECC, i.e. north of 3° S and along 40°–55° E) and along a thin filament near the Kenyan coast (~ 0.5 mg/m^3^ in the model and ~ 0.4 mg/m^3^ in observations) and Tanzanian coast (~ 0.4 mg/m^3^ in the model and ~ 0.3 mg/m^3^ in observations). As for SST, the modelled and satellite data display similar thermal structures with a marked front separating colder waters (< 26.5 °C) concentrated in the area of the most elevated *Chl-a* (i.e. north of 3° S and along 40°–55° E) from the warmer waters (> 29 °C) further south of the SECC. Cooler waters (~ 28 °C) are also observed and correctly modelled as a thin filament near the Kenyan and Tanzanian coasts. The modelled surface currents reproduce the altimetry-derived circulation features well with a clear westward NEMC, northward EACC, eastward SECC and the reversing southward SC. The difference in the magnitude of the current speed (0.8 m/s in the model against 0.6 m/s in altimetry) is because altimetry derived velocity resolves mainly the geostrophic component. The geostrophic limitation also results in a less marked SC flow (from − 1 to 2° N) visible near the equator in the altimetry fields even with their improved processing that uses the β plane approximation^73^.

How well the model simulates the main monsoon variations is shown by comparing the Northeast monsoon patterns to the Southeast monsoon ones. Both the model and satellite data show a similar increase in *Chl-a* concentrations (by ~ 0.2 mg/m^3^) and decrease in SST (by ~ 2 °C) from 40° to 55° E during the Southeast monsoon relatively to the other season. The spatial coverage of the highest *Chl-a* open ocean waters (> 0.65 mg/m^3^ in the model and > 0.45 mg/m^3^ in observations) in open waters extends over a similarly wide area located between − 6° to 12° N and 42° to 56° E. There is also a distinct coastal band near Tanzania and Kenya with higher *Chl-a* (0.5 mg/m^3^ in the model and 0.4 mg/m^3^ in satellite observations) than the immediate offshore waters (~ 0.3 mg/m^3^).

Other notable differences between satellite and modelled data concerns the *Chl-a* concentration near the coasts. The highest *Chl-a* concentrations are captured in the model from south of Zanzibar (7° S) to southern Kenya (3° S), which excludes the area between Mafia and Zanzibar Islands (6.5°–7.5° S) when compared to satellite observations (cf. Fig. 4d,e and Supplementary Fig. S4d, e). This is where the Rufiji river discharges sediments and *Chl-a* rich detritus likely influences high satellite *Chl-a* levels (cf. above in “Methods”), in contrast with the model output, which does not include riverine nutrient and sediment influences.

Modelled SST is simulated accurately compared to observations with coolest waters (< 25 °C) over the Tanzanian / Kenyan coastal band and also north of Madagascar. Finally, modelled and observed NEMC, EACC and SC velocities all accelerate substantially during the Southeast monsoon by more than 0.5 m/s compared to their Northeast monsoon speed. However, the reduced velocities in altimetry (0.9 m/s) relative to the model (1.3 m/s) are likely caused by the geostrophic approximation, which impacts also the SC near the equator.

Overall, the agreement between remote sensing observations and the model lead to high confidence in use of the model output for the analysis of mechanisms affecting *Chl-a* variability in East African waters.

### Lagrangian experiments

The two Lagrangian particle back-tracking experiments used in the study were performed offline, using the ARIANE particle-tracking software^41^ to advect particles using pre-calculated 5-day mean currents from the ORCA0083‐N006 run of the 1/12° NEMO ocean model. Particles were seeded along the Kenyan and Tanzanian coastlines (taken to be the region within 15 km of the coast, as in Popova et al.^42^) at a density of ~ 1 particle per 10 km^2^ (9 particles per model grid cell). Particles were initially seeded at the surface—though not constrained to remain there—and then tracked backwards to their sources 100 days upstream of their seeding locations.

ARIANE interpolates the NEMO velocity field to solve for particle translation through model grid cells, and advects the particles backwards accordingly, with particle positions output at daily frequency. The main advantage of such an approach is that, compared to online passive tracers, it is computationally inexpensive. However, this comes at the cost of not including convection or diffusion and only influence of advection remains.

### Fisheries records

We used the fisheries landings (pure catches) in metric tons of wet weight per year of “herrings, shads and anchovies” recorded within the Tanzanian EEZ over the period 1997–2014 and emanating from the subsistence fishing sector as it is a significant provider of small pelagic fish^10,11^. This dataset is made available by the *Sea Around Us database* (https://www.seaaroundus.org/; see Lam et al.^74^). The *Sea Around Us* project produces reconstructed catches following the method described in Pauly and Zeller^64^ which considers the catch that was “reported” officially to the Food and Agriculture Organization (FAO) or at the national level and estimates of “unreported” catch.

Noteworthy is the fact that official landings, distributed by databases like *Sea Around Us*, are the only data on Tanzanian small pelagic fish catch in open access. Whilst this type of data may not necessarily represent stock abundance due to the absence of fishing effort impact, recent studies^75^ evidenced the consistency of FAO landing trends with those of biomass from fully assessed stocks. The FAO has also officially acknowledged catch reconstructions such as those of *Sea Around Us* help fill the gaps in national fisheries data and demonstrate how catches have realistically changed over time^76^. Furthermore, the latter data have been used to derive the interplay between the environmental variability (such as *Chl-a* concentrations, phytoplankton phenology and SST) and marine fish responses^77^ in different coastal regions like those of Tanzania and Kenya^30^ and Senegal^50^.

## Supplementary information

Supplementary file 1

## References

[1] Van der Elst RP (2009). Nine nations, one ocean: a benchmark appraisal of the South Western Indian Ocean Fisheries Project (2008–2012). Ocean. Coast. Manag..

[2] Jury MR, Enfield DB, Mélice JL (2002). Tropical monsoons around Africa: Stability of El Niño-Southern Oscillation associations and links with continental climate. J. Geophys. Res..

[3] IPCC. Climate Change 2013: The Physical Science Basis. Contribution of Working Group I to the Fifth Assessment Report of the Intergovernmental Panel on Climate Change (eds. Stocker, T.F. et al, Cambridge University Press, Cambridge, United Kingdom and New York, NY, USA) 1535 (2013).

[4] UNDP. *Human Development Reports* https://hdr.undp.org/en/composite/HDI (2018).

[5] Taylor SFW, Roberts MJ, Milligan B, Ncwadi R (2019). Measurement and implications of marine food security in the Western Indian Ocean: an impending crisis?. Food. Sec.

[6] FAO. *The state of food security and nutrition in the world 2018 - building climate resilience for food security and nutrition* https://www.fao.org/3/I9553EN/i9553en.pdf (2018).

[7] FAO. *National Fishery Sector Overview - the United Republic of Tanzania* https://www.fao.org/fishery/docs/DOCUMENT/fcp/en/FI_CP_TZ.pdf (2007).

[8] Ochiewo, J. Social and Economic Impacts of Capture Fisheries and Mariculture. In *The Regional State of the Coast Report: Western Indian Ocean* (eds. UNEP & WIOMSA, Nairobi, Kenya) 306–316 (2015).

[9] Bakun, A., Claude R. & Lluch-Cota S. Coastal upwelling and other processes regulating ecosystem productivity in the Western Indian Ocean. In *Large Marine Ecosystems of the Indian Ocean: Assessemt, Sustainbility, and Management* (eds. Shermann et al., Wiley-Blackwell) 103–141 (1998).

[10] Jiddawi NS, Öhman MC (2002). Marine fisheries in Tanzania. Ambio J. Hum. Environ..

[11] Bultel, E., Doherty, B., Herman, A., Le Manach F. & Zeller D. An update of the reconstructed marine fisheries catches of Tanzania with taxonomic breakdown. In *Fisheries catch reconstructions in the Western Indian Ocean, 1950–2010* (eds. Le Manach F. & Pauly D., Fisheries Centre Research Reports, **23**, University of British Columbia) 151–161 (2015).

[12] Francis, J. & Bryceson, I. Tanzanian coastal and marine resources: some examples illustrating questions of sustainable use. in *Lessons learned: case studies in sustainable use* (eds. Ahmed, J., Gland, Switzerland) 76–102 (2001).

[13] UNESCO-IOC. African Oceans and Coasts. In *IOC Information Document* (eds. Odido M. & Mazzilli S., UNESCO Regional Bureau for Science and Technology in Africa, Kenya) 1255 (2009).

[14] UNEP-Nairobi Convention & WIOMSA. The Regional State of the Coast Report: Western Indian Ocean (eds. UNEP & WIOMSA, Nairobi, Kenya) 546 (2015).

[15] Allison EH (2009). Vulnerability of national economies to the impacts of climate change on fisheries. Fish Fish..

[16] Schott F, McCreary JP (2001). The monsoon circulation of the Indian Ocean. Prog. Oceanogr..

[17] Wyrtki, K. *Oceanographic Atlas of the International Indian Ocean Expedition* (National Science Foundation, Washington, D.C., pp 531, 1971).

[18] Swallow JC, Schott F, Fieux M (1991). Structure and transport of the East African Coastal Current. J. Geophys. Res..

[19] Semba, M., Lumpkin, R., Kimirei, .I, Shaghude, Y. & Nyandwi, N. Seasonal and spatial variation of surface current in the Pemba Channel, Tanzania. *PLoS One.* **14**, 1 (2019). 10.1371/journal.pone.0210303 PMC6322782 30615686

[20] Schott, F. A., Xie S.-P. & J. P. McCreary Jr. Indian Ocean circulation and climate variability. *Rev. Geophys.* **47**, 1 (2009).

[21] Birkett, L. Western Indian Ocean Fishery Resources Survey. *Report on the Cruises of RV Professor Mesyatsev, December 1975–June 1976 / July 1977–December 1977*. *Indian Ocean Programme*. Technical Report 26, Rome, FAO (1979).

[22] Ochumba PBO (1983). Oceanographic features along the Kenyan Coast: implications for fisheries management and development.

[23] Ardill, J. D. & Sanders. M. J. *Proceedings of the Seminar to Identify Priorities for Fisheries Management and Development in the Southwest Indian Ocean*. (Albion, Mauritius, September 1991).

[24] Sætersdal, G., Bianchi, G., Strømme, T. & Venema, S. C. *The Dr Fridtjof Nansen programme 1975–1993. Investigations of Fishery Resources in Developing Regions. History of the Programme and Review of Results.* FAO Fisheries Technical Paper 391. Rome, FAO. pp 434 (1999).

[25] Iversen, S.A., Myklevoli, S., Lwiza, k. & Yonazi, J. Tanzanian marine fish resources in the depth region 10−500 m. Investigated by R/V Dr Fridtjof Nansen. *The Proceedings of the Norad-Tanzania Seminar to Review the Marine Fish Stocks in Tanzania, Mbegani, Tanzania, 6–8 March 1984. Tanzania Fisheries Research Institute, Dar es Salaam and Norwegian Agency for International Development, Bergen*. 45–83 (1984).

[26] Giannoulaki, M., Schismenou, E., Pyrounaki, .M-M & Tsagarakis, K. Habitat Characterization and Migrations, in *Biology and Ecology of Sardines and Anchovies* (eds. Ganias, K., CRC Press, Boca Raton, FL) 190–241 (2014).

[27] Varela R, Álvarez I, Santos F, DeCastro M, Gómez-Gesteira M (2015). Has upwelling strengthened along worldwide coasts over 1982–2010?. Sci. Rep..

[28] Bendekovic, J. & Vuletic, D. Piracy influence on the shipowners and insurance compagnies. in *DAAAM International Scientific Book*. (eds. Katalinic, B. & Tekic Z., Ch. 42, DAAAM Int., Vienna) 711–718 (2013).

[29] Barlow R (2011). Phytoplankton production and adaptation in the vicinity of Pemba and Zanzibar islands Tanzania. Afr. J. Mar. Sci..

[30] Jury M, McClanahan T, Maina J (2010). West Indian Ocean variability and East African fish catch. Mar. Environ. Res..

[31] Anderson, J. & Samoilys, M. The small pelagic fisheries of Tanzania. in *Case Studies on Climate Change and African Coastal Fisheries: A Vulnerability Analysis and Recommendations for Adaptation Options*. FAO Fisheries and Aquaculture Circular 1113 (eds Anderson. J. & Andrew T. pp 19–60, 2016).

[32] Hameed SN, Jin D, Thilakan V (2018). A model for super El Niños. Nat. Commun..

[33] Currie JC (2013). Indian Ocean Dipole and El Niño/Southern Oscillation impacts on regional chlorophyll anomalies in the Indian Ocean. Biogeosciences.

[34] Jacobs, Z.L. et al. Shelf-break upwelling and productivity over the North Kenya Banks: the importance of large-scale ocean dynamics. *J. Geophys. Res*. **125**(1), e2019JC015519 (2020).

[35] Risien CM, Chelton DB (2008). A global climatology of surface wind and wind stress fields from eight years of QuikSCAT scatterometer data. J. Phys. Oceanogr..

[36] Oke PR, Middleton JH (2000). Topographically induced upwelling off eastern Australia. J. Phys. Oceanogr..

[37] Pitcher GC, Figueiras FG, Hickey BM, Moita MT (2010). The physical oceanography of upwelling systems and the development of harmful algal blooms. Prog. Oceanogr..

[38] MacFadyen A, Hickey BM, Foreman MGG (2005). Transport of surface waters from the Juan De Fuca eddy region to the Washington coast. Cont. Shelf Res..

[39] Gallienne CP, Smythe-Wright D (2005). Epipelagic mesozooplankton dynamics around the Mascarene Plateau and Basin, Southwest Indian Ocean. Philos. Trans. R. Soc. Lond. A.

[40] Blanke B, Raynaud S (1997). Kinematics of the Pacific equatorial undercurrent: an Eulerian and Lagrangian approach from GCM Results. J. Phys. Oceanogr..

[41] Popova E (2019). Ecological connectivity between the areas beyond national jurisdiction and coastal waters: Safeguarding interests of coastal communities in developing countries. Marine Policy..

[42] Jury MR, Huang B (2004). The Rossby wave as a key mechanism of Indian Ocean climate variability. Deep Sea Res. Part.

[43] Garrido, S. & Van der Lingen, C.D. Feeding biology and ecology. In *Biology and Ecology of Sardines and Anchovies* (eds. Ganias, K., CRC Press, Boca Raton, FL) 122–189 (2014).

[44] Brochier T (2018). Complex small pelagic fish population patterns arising from individual behavioral responses to their environment. Prog. Oceanogr..

[45] Tsikliras AS, Antonopoulou E, Stergiou KI (2010). Spawning period of Mediterranean marine fishes. Rev. Fish Biol. Fisheries.

[46] Wootton, R.J. & Smith, C. Reproductive biology of teleost fishes (Wiley-Blackwell, New Jersey, pp 496 2014).

[47] Losse, G. F. The Elopoid and Clupeoid fishes of East Africa Coastal waters. *J. East Afr. Nat. Hist. Soc. Natl Mus*. **1**, 77–116 (1968).

[48] Froese, R. & Pauly. D. Clupeidae, Engraulidae. In *FishBase*. (2019).

[49] Lasker, R. The role of a stable ocean in larval fish survival and subsequent recruitment. in *Marine Fish Larvae, Morphology, Ecology and Relation to Fisheries*. (University of Washington, Press: Seattle, WA, USA, pp 80–87 1981).

[50] Kassi JB (2018). Remotely sensing the biophysical drivers of *Sardinella aurita* variability in Ivorian Waters. Remote Sens..

[51] Goes JI, Thoppil PG, Gomes DR (2005). Warming of the Eurasian landmass is making the Arabian Sea more productive. Science.

[52] Roxy MK (2016). A reduction in marine primary productivity driven by rapid warming over the tropical Indian Ocean. Geophys. Res. Lett..

[53] Krishnan R (2006). Indian Ocean-monsoon coupled interactions and impending monsoon droughts. Geophys. Res. Lett..

[54] Annamalai, H., & R. Murtugudde. Role of Indian Ocean in regional climate variability, in *Earth’s Climate: The Ocean-Atmosphere Interaction* (eds. Wang, C., Xie, S.-P. & Carton, J. A., AGU, Washington, D. C). *Geophys. Monogr. Ser.* **147**, 213–246 (2005).

[55] Bopp L (2013). Multiple stressors of ocean ecosystems in the 21st century: projections with CMIP5 models. Biogeosciences.

[56] Racault MF (2017). Impact of El Nino variability on oceanic phytoplankton. Front. Mar. Sci..

[57] Jackson T, Sathyendarnath S, Melin F (2017). An improved optical classification schemes the Ocean Colour Essential Climate Variable and its applications. Remote Sens. Environ..

[58] IOCCG. Remote sensing of ocean colour in coastal and other optically complex waters. In *Reports of the International Ocean Colour Coordinating Group Number 3* (eds. Sathyendrannath, Dartmouth, Canada) 140 (2000).

[59] Chauka LJ (2012). Diversity of the symbiotic Alga Symbiodinium in Tanzanian Scleractinian Corals. Western Indian Ocean J. Mar. Sci..

[60] Chauka LJ, Steinert G, Mtolera MSP (2016). Influence of local environmental conditions and bleaching histories on the diversity and distribution of Symbiodinium in reef-building corals in Tanzania. Afr. J. Mar. Sci..

[61] Zvuloni A, Van Woesik R, Loya Y (2010). Diversity partitioning of stony corals across multiple spatial scales around Zanzibar Island Tanzania. PLoS ONE.

[62] Wagner, G.M. & Sallema-Mtui, R. The Rufiji Estuary: climate change, anthropogenic pressures, vulnerability assessment and adaptive management strategies. In *Estuaries: a lifeline of ecosystem services in the Western Indian Ocean* (eds. Diop, S., Scheren, P. & Machiwa, J.F., Cham, Springer) 183–207 (2016).

[63] Okoola RE (1999). A diagnostic study of the Eastern Africa monsoon circulation during the Northern Hemisphere spring season. Int. J. Climatol..

[64] Pauly D, Zeller D (2016). Catch reconstructions reveal that global marine fisheries catches are higher than reported and declining. Nat. Commun..

[65] Chuenpagdee R, Liguori L, Palomares MD, Pauly D (2006). Bottom-up, global estimates of small-scale marine fisheries catches. Fish. Cent. Res. Rep..

[66] Scott LEP (2004). Information Management and Environmental Education Report: ACEP Seventh Expedition ALG 130.

[67] ACEP. ACEP Seventh Expedition ALG 130. *African Coelacanth Ecosystem Programme*, Cruise Report, South Africa, (2004).

[68] Madec, G., & the NEMO team. NEMO ocean engine. Note du Pole de modelisation. Institut Pierre-Simon Laplace (IPSL, France). **27**, 1288–1619, (2008).

[69] Brodeau L, Barnier B, Penduff T, Treguier AM, Gulev S (2010). An ERA40-based atmospheric forcing for global ocean circulation models. Ocean Model..

[70] Dussin R, Barnier B, Brodeau L, Molines J-M (2016). The making of Drakkar forcing set DFS5.

[71] Yool A, Popova EE, Anderson TR (2013). MEDUSA-2.0: an intermediate complexity biogeochemical model of the marine carbon cycle for climate change and ocean acidification studies. Geosci. Model Dev..

[72] Srokosz MA, Robinson J, McGrain H, Popova EE, Yool A (2015). Could the Madagascar bloom be fertilized by Madagascan iron?. J. Geophys. Res..

[73] Pujol MI (2016). DUACS DT2014: the new multi-mission altimeter data set reprocessed over 20 years. Ocean. Sci..

[74] Lam, V.W.Y., Tavakolie, A., Pauly, D. & Zeller, D. The Sea Around Us databases and their spatial dimensions. In *Catch reconstructions: concepts, methods and data sources* (eds. Pauly, D. & Zeller, D., University of British Columbia, 2015).

[75] Froese R, Zeller D, Kleisner K, Pauly D (2012). What catch data can tell us about the status of global fisheries. Mar. Biol..

[76] Pauly D, Zeller D (2018). Agreeing with FAO: comments on SOFIA 2018. Mar. Policy..

[77] Tzanatos E, Raitsos DE, Triantafyllou G, Somarakis S, Tsonis AA (2014). Indications of a climate effect on Mediterranean fisheries. Clim. Change..

