# Supplementary material for: Interannual monsoon wind variability as a key driver of East African small pelagic fisheries
Source: Sci Rep. 2020 Aug 6;10:13247. doi: 10.1038/s41598-020-70275-9 (PMC7413268; doi:10.1038/s41598-020-70275-9)
Supplement: Supplementary file 1 [file 41598_2020_70275_MOESM1_ESM.pdf]

Interannual monsoon wind variability as a key driver of East African small pelagic fisheries

Fatma Jebri[1,*], Zoe Jacobs[1], Dionysios E. Raitsos[2,3], Meric Srokosz[1], Stuart C. Painter[1], Stephen Kelly[1], Mike Roberts[1,4], Lucy Scott[5], Sarah F. W. Taylor[1], Matthew Palmer[1], Hellen Kizenga[6], Yohana Shaghude[6], Juliane Wihsgott[1] and Ekaterina Popova[1]

[1] National Oceanography Centre, Southampton, SO14 3ZH, United Kingdom
[2] Department of Biology, National and Kapodistrian University of Athens, Athens, Greece
[3] Plymouth Marine Laboratory, Plymouth, PL1 3DH, United Kingdom
[4] Nelson Mandela University, Ocean Science and Marine Food Security, Port Elizabeth, 6001, South Africa
[5] South African Environmental Observation Network, Egagasini Node, Cape Town, South Africa
[6] Institute of Marine Sciences, Zanzibar, Tanzania

* corresponding author: fatma.jebri@noc.ac.uk

Supplementary Information (Figures and Text)

This part contains 15 supplementary Figures, and 4 supplementary Texts

Supplementary Figure S1:

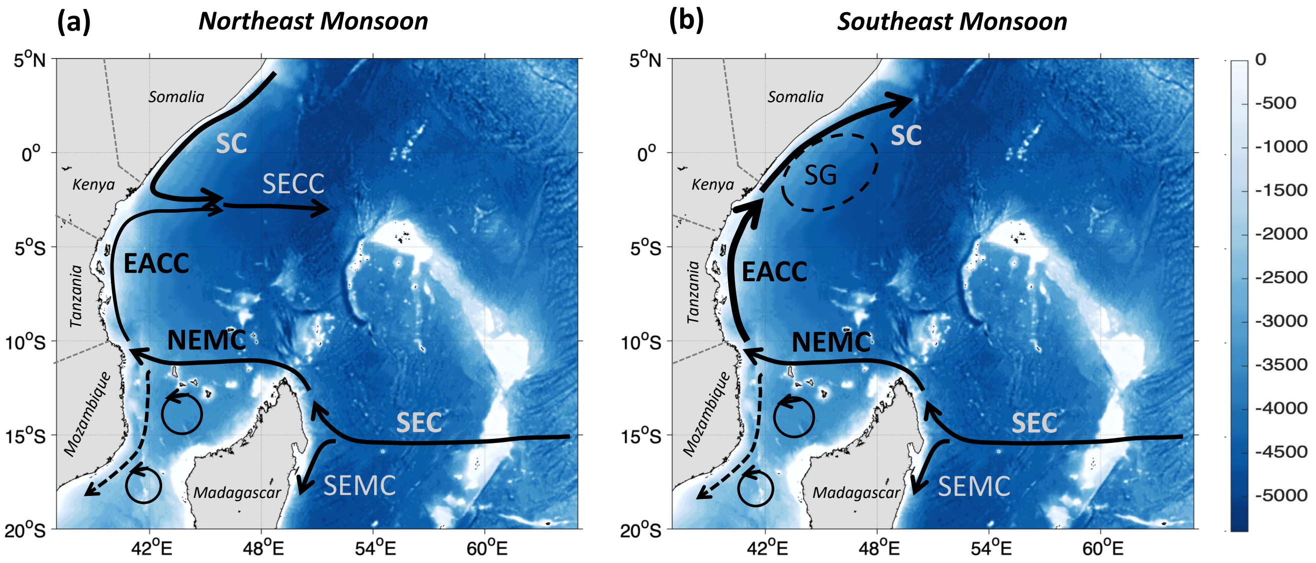

Figure S1: Schematic view of the major ocean circulation features based on Jacobs et al. [34] ***and bathymetry (in m) over the Western Indian Ocean (WIO) during the (a) Northeast monsoon and the (b) Southeast monsoon.*** *From East to West - SEC: South Equatorial current, SEMC: Southeast Madagascar Current, NEMC: Northeast Madagascar Current, EACC: East African Coastal Current, SC: Somali Current, SG: Southern Gyre and SECC: Southeast Counter Current. The thickness of the EACC arrow indicates its seasonal strengthening. Maps on panels (a) and (b) were created by the authors using MATLAB software vR2015b (see https://uk.mathworks.com/products/new_products/release2015b.html and https://uk.mathworks.com/products/matlab.html).*

Supplementary Text 1:

Choice of region for analysis of satellite Chl-a in Fig. 2

The region chosen for the spatiotemporal analysis of satellite *Chl-a* presented in Fig. 2 is as follow:

- Setting the eastern boundary of the black box at 40.15˚E in order include the entire islands East-coast production areas.

- Including the shallow areas between the islands and the mainland coastline as suspended materials are low due to the abundance of coral reefs [59,60,61].

- Excluding an area around the Rufiji River outflow due to the significant amount of suspended material[62]. The Rufiji outflow area is excluded using a mask limited by the lines of latitude between the coast and Mafia Island, north and south of the river mouth (See white mask on Fig. 2a). Testing the sensitivity by varying the mask size, had little effect on the averaged *Chl-a* timeseries (small change in the magnitude of Chl-a timeseries but no difference in the overall behaviour).

<u>Supplementary Figure S2</u>:

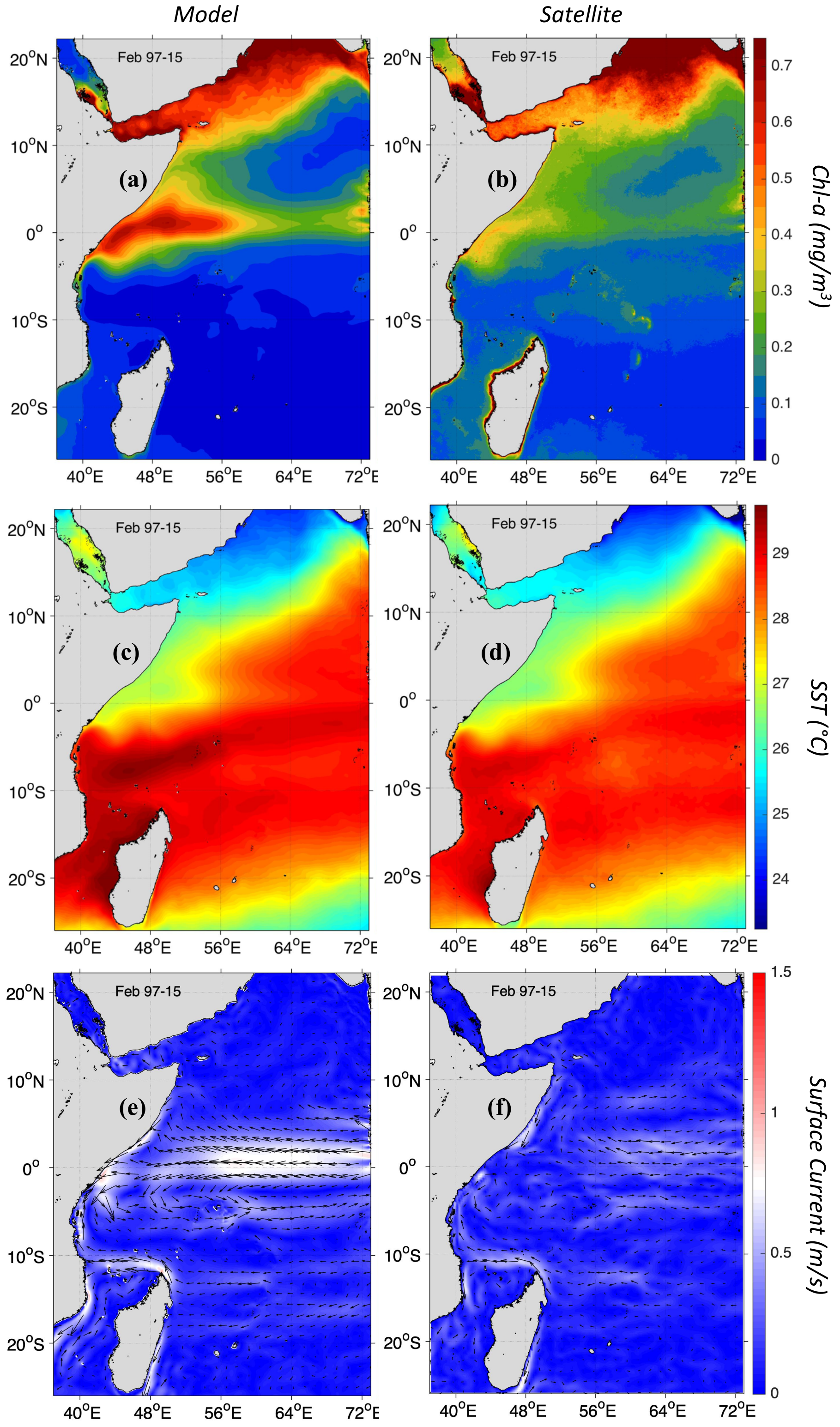

Figure S2: Validation of the model outputs over the WIO for climatological February (Northeast monsoon) over the period 1997-2015. *Surface Chl-a in mg/m^3 derived from modelled* ***(a)*** *and satellite data* ***(b)****, SST in °C from modelled* ***(c)*** *and satellite data* ***(d)****, and surface currents in m/s from modelled* ***(e)*** *and satellite data* ***(f)****. The model current vectors are displayed every 2 grid points to be consistent with the lower resolution of satellite altimetry currents. Maps on all panels were created by the authors using MATLAB software vR2015b (see*

https://uk.mathworks.com/products/new_products/release2015b.html and https://uk.mathworks.com/products/matlab.html).

Supplementary Figure S3:

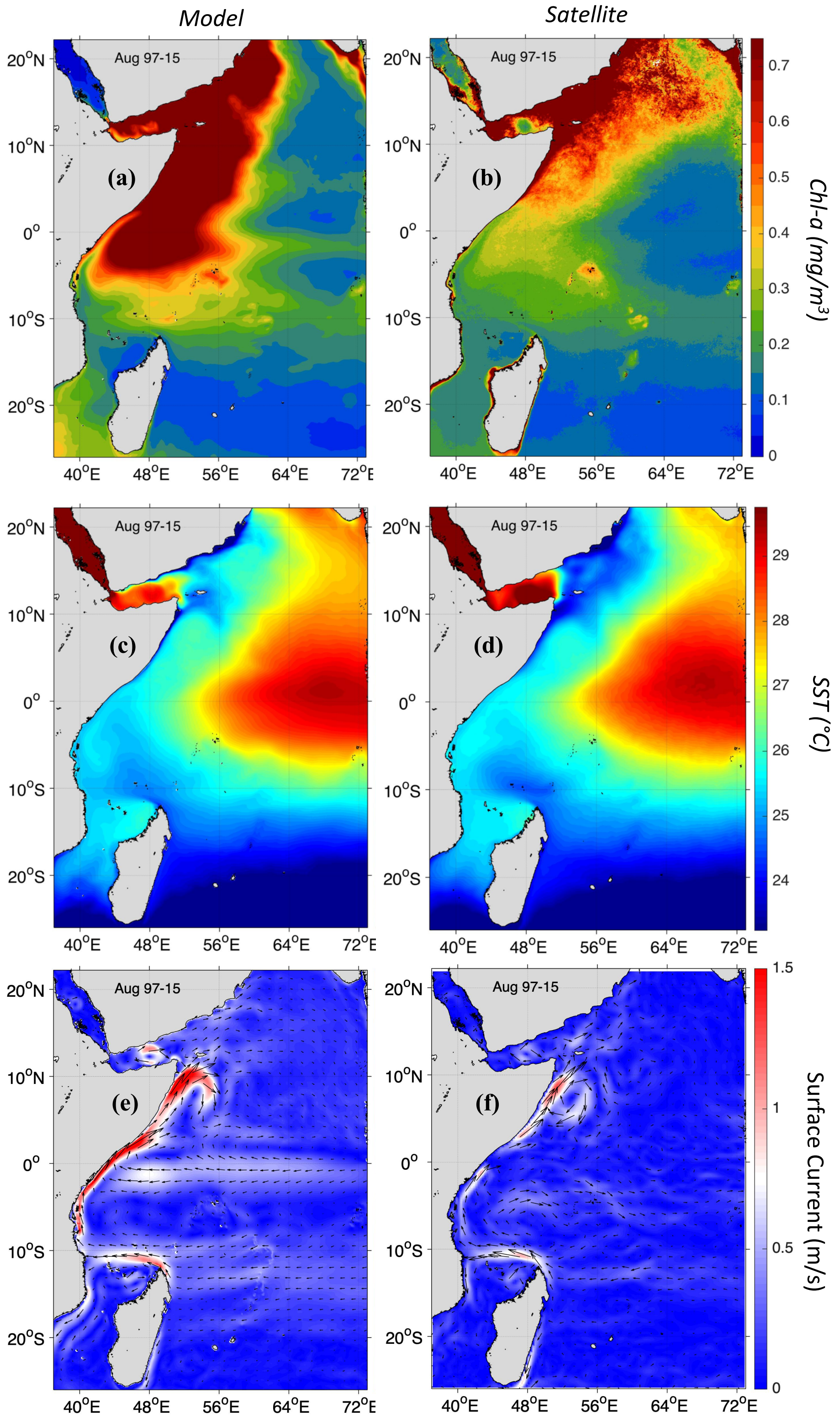

Figure S3: Validation of the model outputs over the WIO for climatological August (Southeast monsoon) over the period 1997-2015. *Surface Chl-a in mg/m^3 derived from modelled* ***(a)*** *and satellite data* ***(b)****, SST in °C from modelled* ***(c)*** *and satellite data* ***(d)****, and surface currents in m/s from modelled* ***(e)*** *and satellite data* ***(f)****. The model current vectors are displayed every 2 grid points to be consistent*

with the lower resolution of satellite altimetry currents. Maps on all panels were created by the authors using MATLAB software vR2015b (see https://uk.mathworks.com/products/new_products/release2015b.html and https://uk.mathworks.com/products/matlab.html).

<u>Supplementary Figure S4</u>:

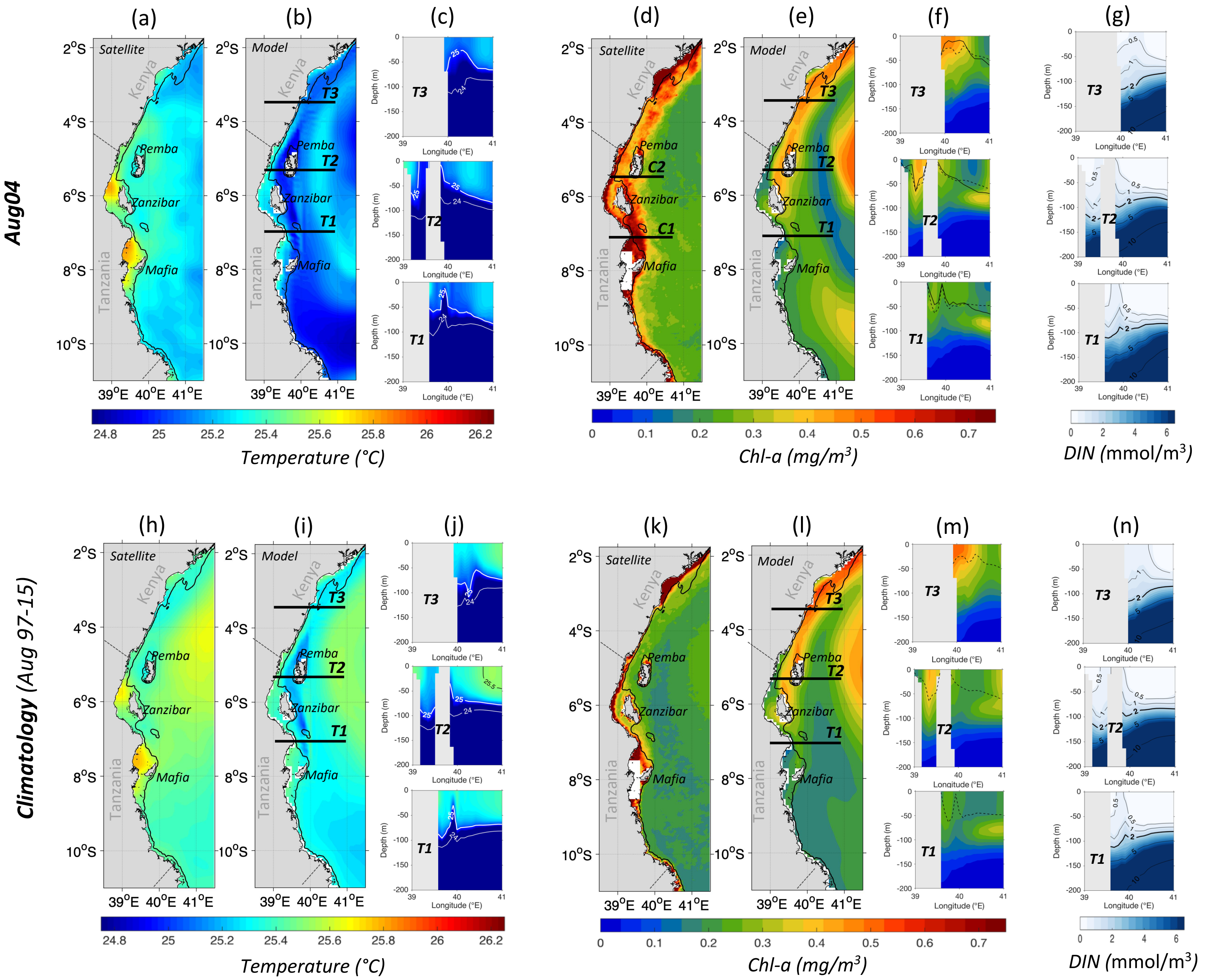

Figure S4: Surface and subsurface signatures of dynamic uplift upwelling along the Tanzanian and Kenyan coasts during Aug04 relatively to the climatology (1997-2015). *SST in °C from satellite data* ***(a, h)*** *and the model* ***(b, i)*** *and surface Chl-a in mg/m^3 derived from satellite data* ***(d, k)*** *and the model* ***(e, l)****. Satellite Chl-a data on the Rufiji river outflow area are masked in white on panels (d) and (k). The 200m isobath derived from ETOPO2v1 global gridded database are represented by solid and dashed black lines respectively. Cross-sections (T1 to T3) of modelled temperature in °C* ***(c, j)****, Chl-a in mg/m^3* ***(f, m)*** *and DIN in mmol/m^3* ***(g, n)*** *are displayed along three locations as indicated on panels* ***(b)****,* ***(e)****,* ***(i)*** *and* ***(l)*** *with back horizontal lines. The MLD in m of Aug04 and the climatology are represented by the black solid and dashed lines respectively on panel* ***(f)****. The 2 mmol/m^3 isopleth is highlighted with a thick black line on panels g and n. Note that black horizontal lines on the satellite*

Chl-a panel ***(d)*** *represent the CTD transects locations examined on Figure S4. Maps on panels (a), (b), (d), (e), (h), (i), (k) and (l) were created by the authors using MATLAB software vR2015b (see* *https://uk.mathworks.com/products/new_products/release2015b.html* *and* *https://uk.mathworks.com/products/matlab.html*).

Supplementary Text 2:

Strong dynamic uplift upwelling along Tanzanian and Kenyan coasts in Aug04

A similar to Sep02, but less pronounced situation is observed in Aug04 relative to the 19-year climatological mean (1997-2015) (Fig. S4, a, d, h and k). The satellite data reveals a coastal band of elevated *Chl-a* (up to 0.75 mg/m^3) from Mafia Island (8.5°S) to Kenya (1°S), contrasting with the waters further East with low *Chl-a* (down to 0.3 mg/m^3) (Fig. S4 d and k). These *Chl-a* concentrations intensified in Aug04 by about 1.5 times their climatological mean (Fig. S4 d and k). The observed SST have cooled down to 25.1°C as compared to ~ 25.5°C in a normal August (Fig. S4a and h). In the model, Aug04 *Chl-a* is less well resolved than in Sep02 (cf. Fig. 4e) with maximum values not exceeding 0.45 mg/m^3 (Fig. S4e). But in the model Aug04 shows high *Chl-a* concentrations along the coasts of Tanzania and Kenya, in agreement with remote sensing. The modelled SST also shows anomalously low values, decreasing from 25.2°C in normal conditions to below 24.7°C for Aug04, over a similar spatial extent as in the satellite observations (Fig. S4b and i).

There is a clear pattern of high *Chl-a* concentrations associated with cool waters along the coastline during Aug04, as the case of Sep02 indicating an intensified upwelling or enhanced vertical mixing signal. The fact that the MLD in Aug04 across three vertical crossections along the elevated *Chl-a* coastal band between Mafia and Kenya (T1 to T3, see Fig. S4e for exact positions) is shallower than the climatology (Fig. S4f), suggests that the interannual *Chl-a* intensifications are not caused by enhanced mixing but likely by upwelling.

The upwelling subsurface signature can be assessed in the modelled temperature, *Chl-a*, Dissolved Inorganic Nitrogen (DIN) of sections T1 to T3. In a climatological August, the sections T1 to T3 indicate cool SST, high *Chl-a* and elevated nutrients near the coast (Fig. S4j, m and n), typical of an upwelling regime. The 25°C isotherm rises around 39.5°E from ~125m to 40-50 m on sections T1-T3. This uplifts the 25°C isotherms on sections T1 to T3 in the climatological August. These cold temperatures are accompanied by high *Chl-a* concentrations of 0.4-0.5 mg/m^3 in the upper 80 m. The elevated *Chl-a* is consistent with the doming of DIN isopleths on sections T1 to T3 near the coasts. During Aug04, the same situation occurs with more intensity (Fig. S4c, f and g). The cold waters with elevated nutrients are closer to the surface than in the climatological year, leading to higher *Chl-a* concentrations (Fig. S4f and m). The elevated *Chl-a* over 80 m depth is around 0.45 mg/m^3 in Aug04. The 25°C isotherm in Aug04 is uplifted to 20-30 m on sections T1 and T3 and outcrops completely on section T2 (Fig. S4c). Elevated *Chl-a* concentrations of around 0.5 mg/m3 are visible over the upper 80 m in Aug04 (Fig. S4f). The prominent *Chl-a* signal near the coasts coincides with a more accentuated doming of the 2 mmol/m^3 DIN isopleth for Aug04 than the climatology (Fig. S4g).

Supplementary Figure S5:

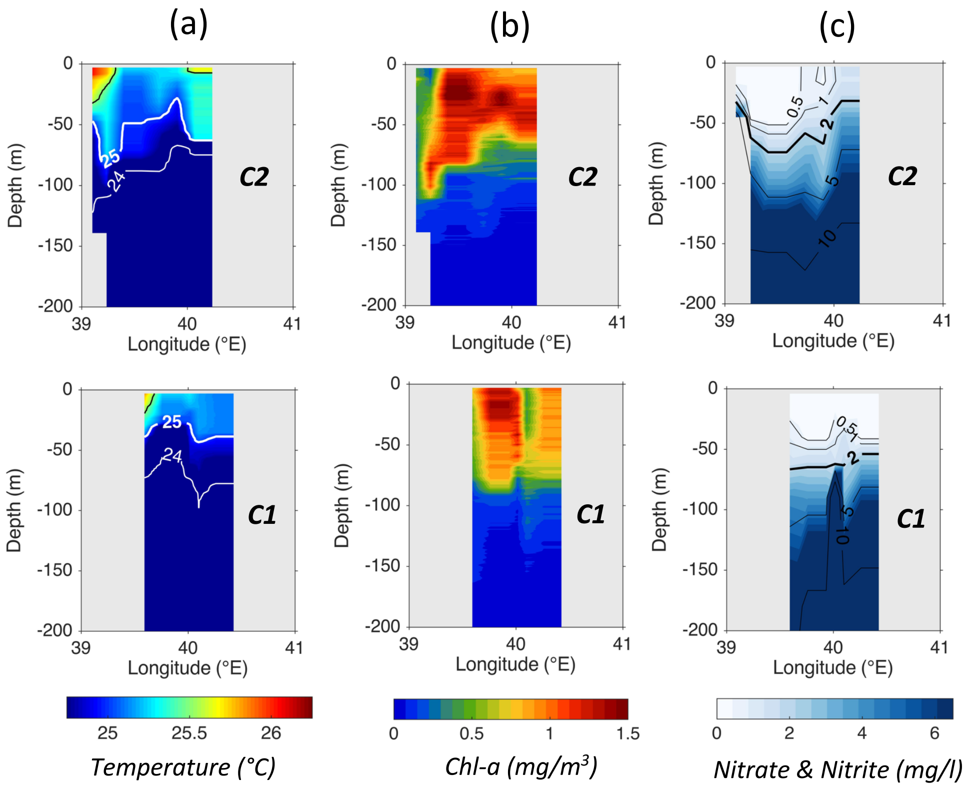

Figure S5: Coincidental in-water measurements of biophysical parameters during the Aug04 bloom at Tanzanian waters*. CTD cross-sections (C1 to C2) of temperature in °C* ***(a)****, fluorescence in mg/m*^{3} ***(b)****, and Nitrate & Nitrite in mg/l* ***(c)*** *are displayed along three locations as indicated with the back horizontal lines on Fig. S4 (d). Note that these locations are as close as possible (but not exactly) to the model sections T1 and T2. The 2 mmol/m*^{3} *isopleth is highlighted with a thick black line on panel* ***(c)****.*

Supplementary Text 3:

Quality of simulated upwelling signature (temperature, Chl-a, DIN) at Tanzanian waters

The modelled 2004 subsurface pattern is coherent with *in situ* temperature, fluorescence and Nitrate & Nitrite sections sampled from South of Zanzibar to Pemba. The presented *in-situ* cross-sections (C1 and C2) locations are on the same latitude as model sections (T1 and T2) but with a shorter longitudinal extent (see Fig. S4d). The observed temperatures on Fig. S5a show cooler waters of ~25.2°C reaching the surface on sections C1-C2 along the coasts. The thermocline is pushed upwards towards the surface. High levels of fluorescence (a proxy for *Chl-a*) ranging between 1 and 1.5 mg/m^3 are seen on Fig. S5b in the upper 40 m, which signify high phytoplankton biomass along the coastal band from South of Zanzibar to Pemba. This is coherent with the downward sloping of the 2 mg/l Nitrate & Nitrite isopleth (Fig. S5c), which is located at the ~80 m layer during an inter-monsoon period [80], but is now observed at a depth of 50 m, which is within the photic zone.

<u>Supplementary Figure S6</u>:

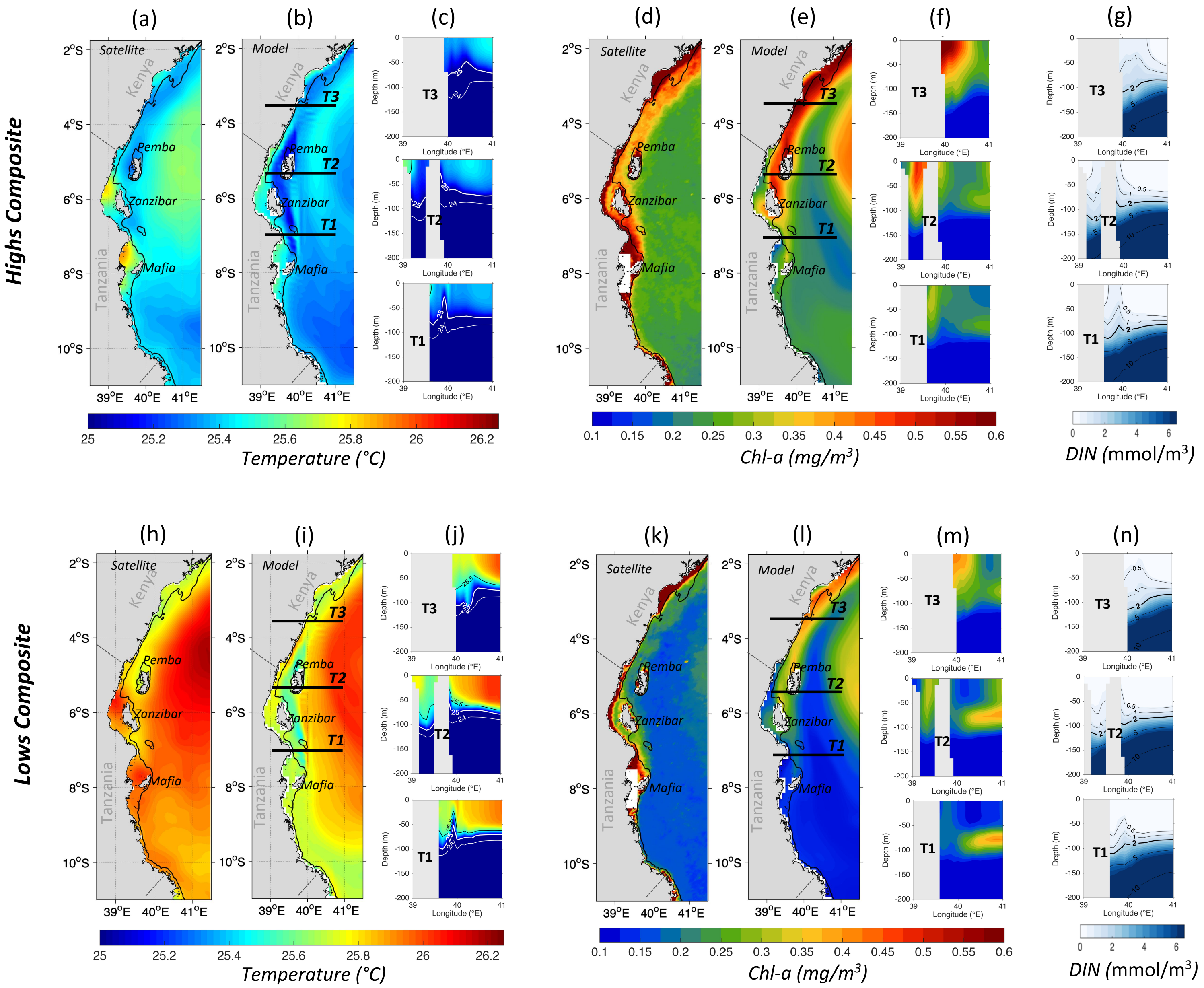

Figure S6: Surface and subsurface signatures of dynamic uplift upwelling along the Tanzanian and Kenyan coasts during for composites of Southeast monsoons Chl-a "highs" and "lows" over the period 1997-2015. *SST in °C from satellite data* ***(a, h)*** *and the model* ***(b, i)*** *and surface Chl-a in mg/m^3 derived from satellite data* ***(d, k)*** *and the model* ***(e, l)****. Satellite Chl-a data on the Rufiji river outflow area are masked in white on panels (d) and (k). The 200m isobath derived from ETOPO2v1 global gridded database are represented by solid and dashed black lines respectively. Cross-sections (T1 to T3) of modelled temperature in °C* ***(c, j)****, Chl-a in mg/m^3* ***(f, m)*** *and DIN in mmol/m^3* ***(g, n)*** *are displayed along three locations as indicated on panels* ***(b)****,* ***(e)****,* ***(i)*** *and* ***(l)*** *with back horizontal lines. The MLD in m of Aug04 and the climatology are represented by the black solid and dashed lines respectively on panel* ***(f)****. The 2 mmol/m^3 isopleth is highlighted with a thick black line on panels g and n. Note that black horizontal lines on the satellite Chl-a panel* ***(d)*** *represent the CTD transects locations examined on Figure S4. Maps on panels (a), (b), (d), (e), (h), (i), (k) and (l) were created by the authors using MATLAB software vR2015b (see https://uk.mathworks.com/products/new_products/release2015b.html and https://uk.mathworks.com/products/matlab.html).*

Supplementary Figure S7:

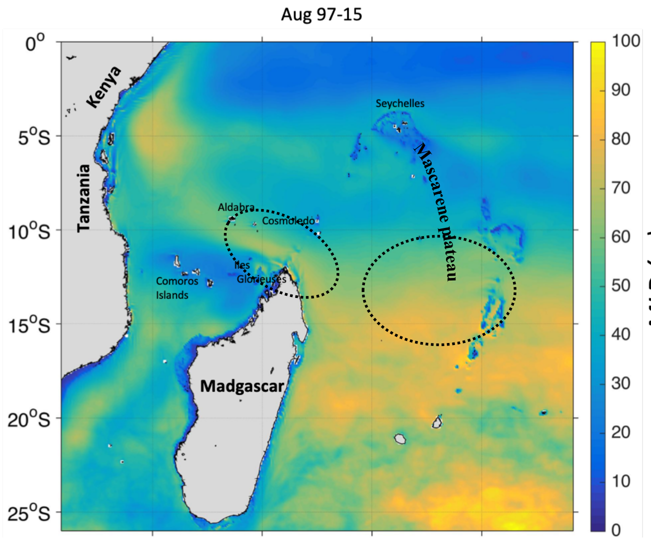

***Figure S7: Modelled MLD in m over the southern WIO during for climatological August (Southeast monsoon) over the period 1997-2015**. Areas of enhanced mixing around Madagascar northern tip and the Mascarene plateau are indicated by the black ellipses. The maps was created by the authors using MATLAB software vR2015b (see https://uk.mathworks.com/products/new_products/release2015b.html and https://uk.mathworks.com/products/matlab.html).*

Supplementary Figure S8:

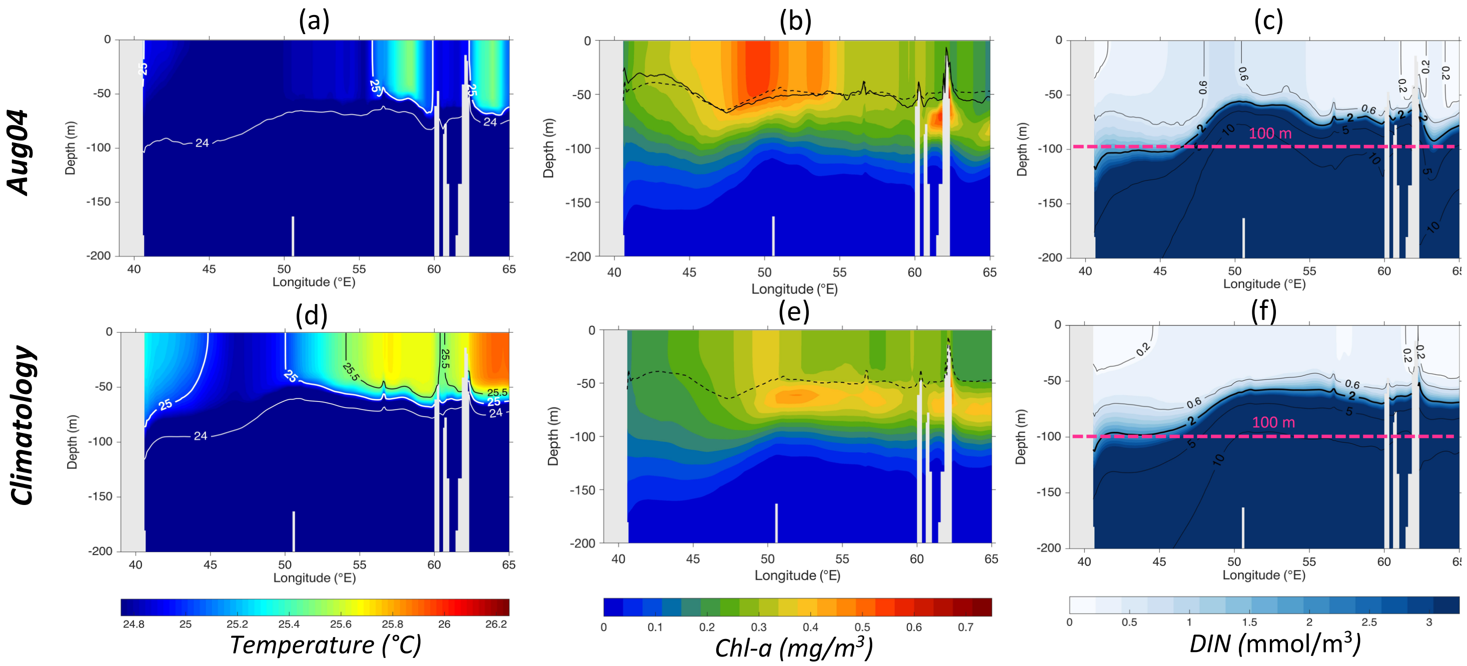

***Figure S8: An Advective impact along the NEMC path of nutrients rich and cold waters from the Madagascar northern tip to the Tanzanian coast during Aug04 and the climatology.** A cross-section (T0) from 39 to 65°E of modelled temperature in °C **(a, d)**, Chl-a in mg/m^3 **(b, e)** and DIN in mmol/m^3 (c, f) is displayed along the latitude line 10°S as indicated with the black line on Fig. 5g. The MLD in m of Aug04 and the climatology are represented by the black solid and dashed lines respectively on panel **(b)**. The 2 mmol/m^3 isopleth is highlighted with a thick black line on panels **(c)** and **(f)**.*

Supplementary Figure S9:

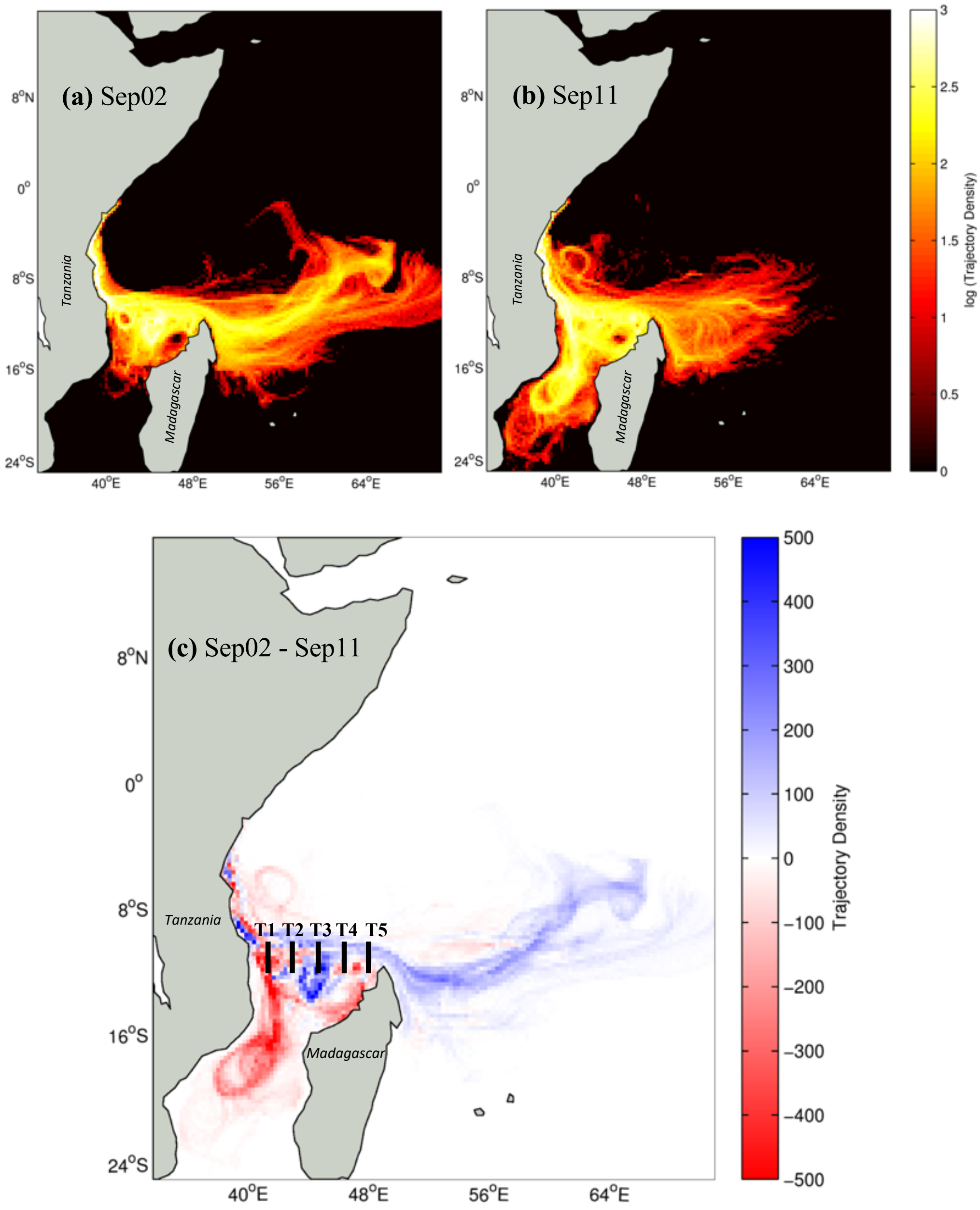

Figure S9: Trajectory densities of virtual particles presented for the first 100 days of backtracking from the East African coastal zones in (a) Sep02 and (b) Sep11, back to their upstream sources in the surface Indian Ocean. *The difference in trajectory density between Sep02 and Sep11 is shown on panel* ***(c)****. The density of trajectories is calculated by dividing the ocean into 0.25° x 0.25° grid cells and counting the number of trajectories timesteps that are recorded in each cell for all particles. Note that black vertical lines on panel* ***(c)*** *represent the cross-sections (T1-T5) locations examined on Figure S9. Maps on all panels were created by the authors using MATLAB software vR2013a (see https://uk.mathworks.com/videos/r2013a-release-highlights-75269.html and https://uk.mathworks.com/products/matlab.html).*

Supplementary Figure S10:

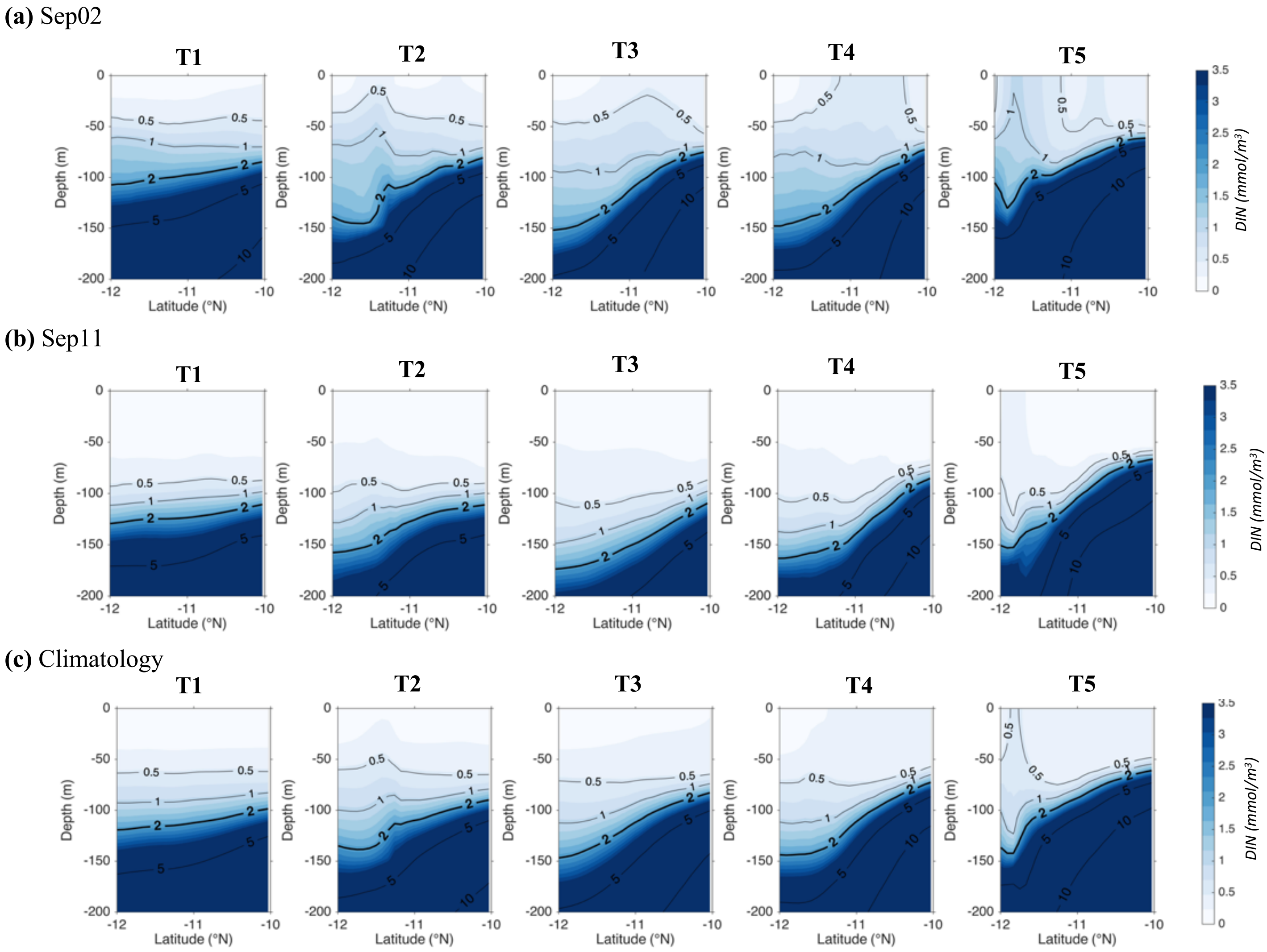

Figure S10: Cross-sections (T1 to T5) of modelled DIN in mmol/m^3 across the path of the NEMC at 5 locations (41°E; 43°E; 45°E; 47°E and 49°E) as indicated on panel (c) with the back vertical lines, during (a) Sep02, (b) Sep11 and (c) the climatology. *The 2 mmol/m^3 isopleth is highlighted with a thick black line on panels g and n.*

Supplementary Figure 11:

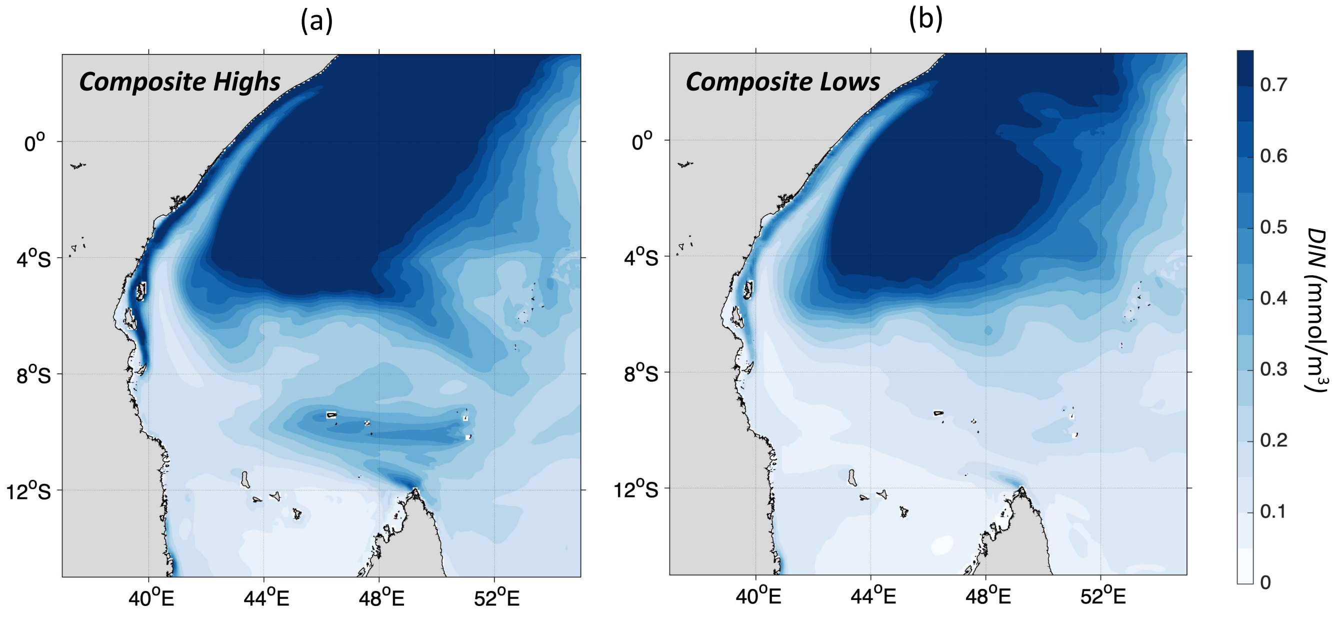

Figure S11*: Nutrient rich waters over the southern WIO for composites of Southeast monsoons Chl-a "highs" and "lows" over the period 1997-2015.* *Modelled Surface DIN in mmol/m^3 for* ***(a)*** *Composite "highs" and* ***(b)*** *Composite "lows". Maps on panels (a) and (b) were created by the authors using MATLAB software vR2015b (see https://uk.mathworks.com/products/new_products/release2015b.html and https://uk.mathworks.com/products/matlab.html).*

Supplementary Text 4:

Differences of the ocean conditions between the high and low catch years

To further highlight how different are the biophysical ocean conditions during the Southeast monsoon of the high and low catch years, we assess two composites of modelled (Fig. S12) and remotely sensed (Fig. S13) SST, Chl-a and surface current speeds constructed as high minus low catch years following Jury et al.,[30] method. Composite 1 (2002-2011) obtained from the model and satellite data show a pronounced increase in Chl-a along the East African coastal band. This is associated with stronger surface current speeds over the NEMC and EACC paths and a large-scale cooling over the WIO Indian ocean. Composite 2 (2005-2011) show similar patterns as Composite 1 for both model and satellite data, but less pronounced.

Supplementary Figure S12:

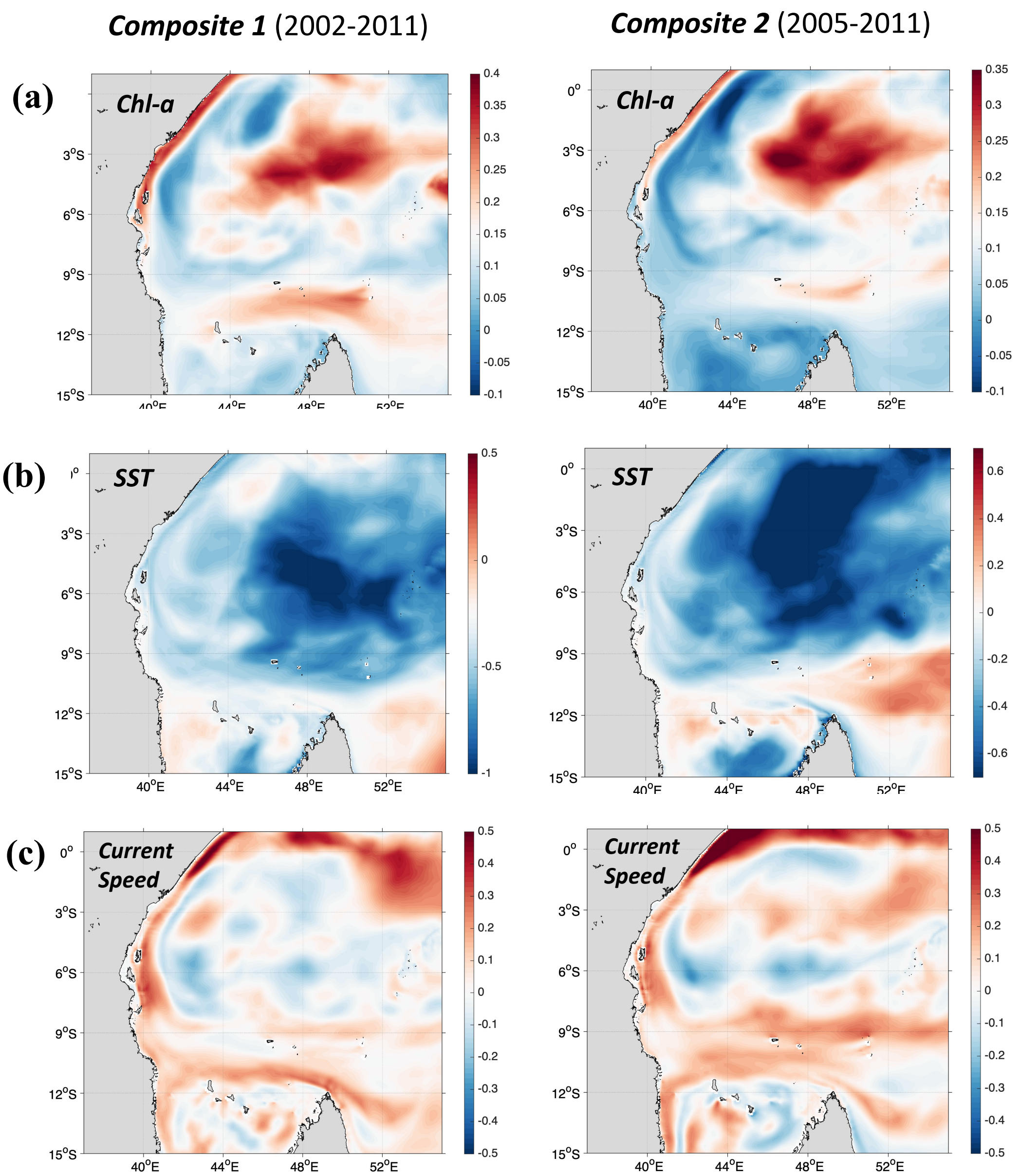

Figure S12: Composite maps of modelled (a) Chl-a, (b) SST and (c) Current Speed during the Southeast monsoon, constructed as differences between the high minus low catch years. *Composite 1 (2) represent the high catch of 2002 (2005) minus the low catch of 2011(2011). Maps on all panels were created by the authors using MATLAB software vR2015b (see https://uk.mathworks.com/products/new_products/release2015b.html and https://uk.mathworks.com/products/matlab.html).*

__Supplementary Figure S13:__

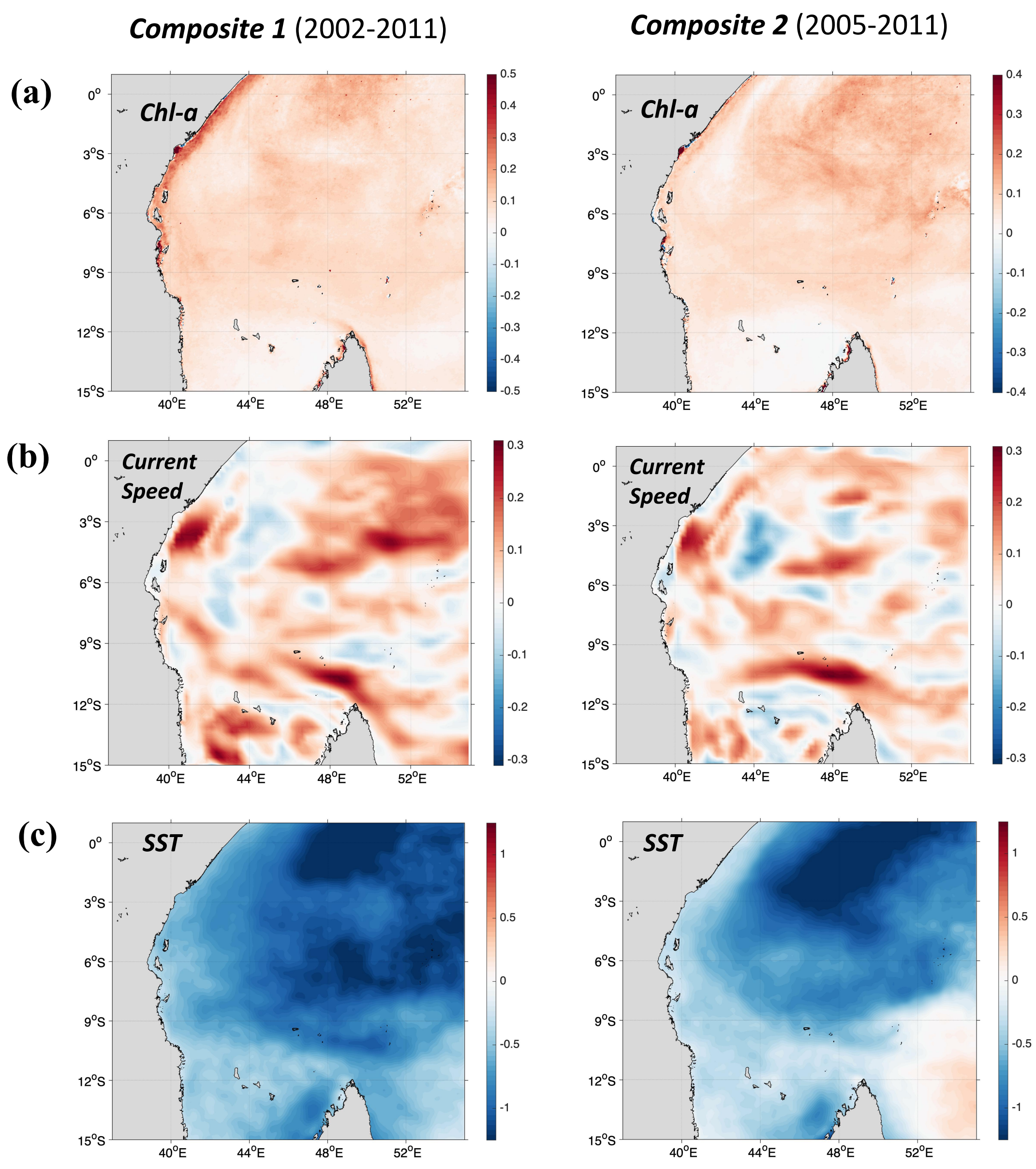

Figure S13: Same as Figure S12 but from satellite data. *Maps on all panels were created by the authors using MATLAB software vR2015b (see https://uk.mathworks.com/products/new_products/release2015b.html and https://uk.mathworks.com/products/matlab.html).*

Supplementary Figure S14:

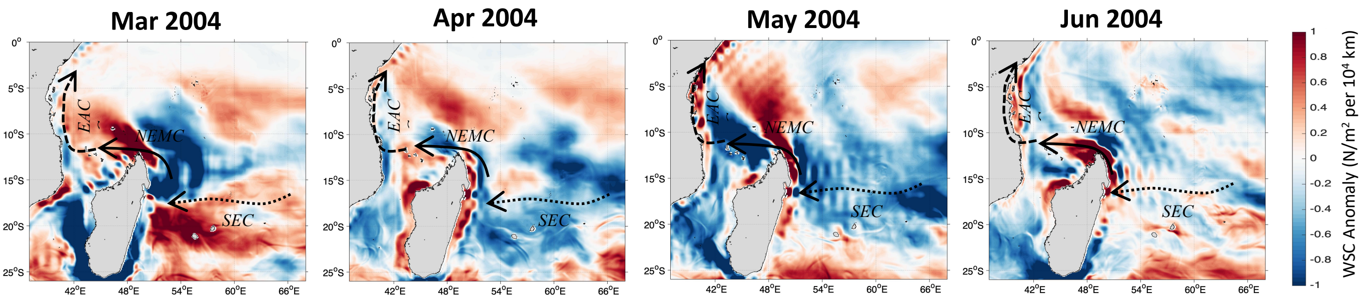

Figure S14: Wind Stress Curl (WSC) forcing over the southern WIO from March to June 2004. *Wind stress vectors in (a) are displayed every 25 grid points. WSC anomalies from March to June **(b)** 2002 and **(c)** 201, relatively to the period 1997-2015. A schematic view of the South Equatorial Current (SEC), North East Madagascar Current (NEMC) and East African Coastal Current (EACC) based on Schott et al.*[20] *is superimposed to show the areas of influence of Southeast winds. Maps on panels (a) to (c) were created by the authors using MATLAB software vR2015b (see https://uk.mathworks.com/products/new_products/release2015b.html and https://uk.mathworks.com/products/matlab.html).*

Supplementary Figure S15:

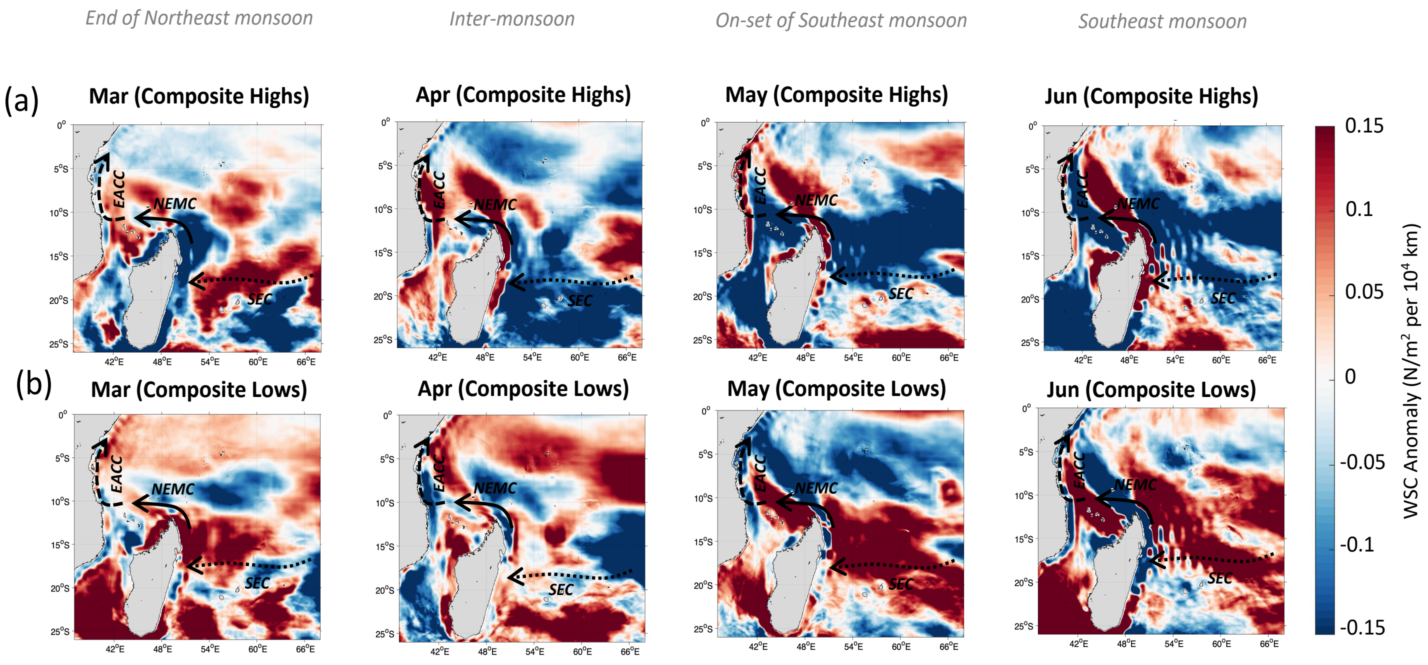

Figure S15: Wind Stress Curl (WSC) Anomalies over the southern WIO from March to June (a) for composites of Southeast monsoons Chl-a "highs" and "lows" over the period 1997-2015. *WSC Anomalies from March to June for **(a)** Composite "highs" and **(c)** Composite "lows". A schematic view of the South Equatorial Current (SEC), North East Madagascar Current (NEMC) and East African Coastal Current (EACC) based on Schott et al.*[20] *is superimposed to show the areas of influence of Southeast winds. Maps on panels (a) to (c) were created by the authors using MATLAB software vR2015b (see https://uk.mathworks.com/products/new_products/release2015b.html and https://uk.mathworks.com/products/matlab.html).*

References

20. Schott, F. A., Xie S.-P. & J. P. McCreary Jr. Indian Ocean circulation and climate variability. *Rev. Geophys.* **47**, (2009).

30. Jury, M., McClanahan, T. & Maina, J. West Indian Ocean variability and East African fish catch. *Marine environmental research*. **70**, 162-170 (2010).

34. Jacobs, Z.L. et al. Shelf-break upwelling and productivity over the North Kenya Banks: the importance of large-scale ocean dynamics. *J. Geophys. Res*. **125**(1), e2019JC015519 (2020).

40. Gallienne, C.P. & Smythe-Wright, D. Epipelagic mesozooplankton dynamics around the Mascarene Plateau and Basin, Southwest Indian Ocean. *Philosophical Transactions of the Royal Society of London A*, **363**. 191-202 (2005).

59. Chauka, L. J. Diversity of the Symbiotic Alga Symbiodinium in Tanzanian Scleractinian Corals. *Western Indian Ocean J. Mar. Sci*. **11**,67-72 (2012).

60. Chauka, L.J., Steinert, G. & Mtolera, M.S.P. Influence of local environmental conditions and bleaching histories on the diversity and distribution of Symbiodinium in reef-building corals in Tanzania. *African Journal of Marine Science*. **38**, 57-64 (2016).

61. Zvuloni, A., Van Woesik, R. & Loya, Y. Diversity Partitioning of Stony Corals Across Multiple Spatial Scales Around Zanzibar Island, Tanzania. *PLoS ONE*. **5,** e9941 (2010).

62. Wagner, G.M. & Sallema-Mtui, R. The Rufiji Estuary: climate change, anthropogenic pressures, vulnerability assessment and adaptive management strategies. In *Estuaries: a lifeline of ecosystem services in the Western Indian Ocean* (eds. Diop, S., Scheren, P. & Machiwa, J.F., Cham, Springer) 183–207 (2016).

80. Roberts, M. J. et al., 2007 Western Indian Ocean Cruise and Data Report - ALG 160, *African Coelacanth Ecosystem Program*, Grahamstown, South Africa (2008).
